# GLUT3 enhances chemosensitivity in glioblastoma by transporting temozolomide and capecitabine

**DOI:** 10.1038/s41420-025-02664-w

**Published:** 2025-08-14

**Authors:** Honglin Diao, Yuxin Sun, Xiaojia Zhou, Qikai Wang, Mingyue Wang, Keyu Chen, Zhihua Huang, Jianlei Wei, Zeping Li, Yaxin Lou, Zebin Mao, Wenhua Yu

**Affiliations:** 1 Key Laboratory of Carcinogenesis and Translational Research (Ministry of Education), Department of Biochemistry and Molecular Biology, School of Basic Medical Sciences, Peking University Health Science Center, Beijing, China; 2 Key Laboratory of Natural and Biomimetic Drugs, School of Pharmaceutical Sciences, Peking University, Beijing, China

**Keywords:** CNS cancer, CNS cancer, Chemotherapy

## Abstract

Glioblastoma multiforme (GBM), the most aggressive brain cancer, is highly resistant to chemotherapy, which profoundly affects patient survival and prognosis. Temozolomide (TMZ), the sole first-line chemotherapeutic agent for GBM, faces substantial challenges in overcoming this resistance. Despite the belief that TMZ is well-absorbed in the small intestine and can effectively cross the blood-brain barrier due to its small molecular size, emerging evidence suggests that its uptake is not merely through passive diffusion across the lipid bilayer but is regulated by Wnt signaling. However, the precise mechanism governing TMZ uptake remains elusive. GLUT3, which is highly expressed in GBM and primarily functions as a glucose transporter, has emerged as a promising therapeutic target. This study demonstrates that GLUT3 upregulation in GBM cells enhances sensitivity to both TMZ and capecitabine (CAPE). Uptake assays revealed that GLUT3 overexpression (OE) or knockdown (KD) significantly influenced the uptake of these chemotherapeutic agents. We further validated the interaction between GLUT3 and TMZ/CAPE through molecular docking, dynamics simulations, and MST assay. Site-directed mutagenesis identified eight amino acids involved in GLUT3-mediated binding and transport of TMZ and CAPE. A mouse xenograft model confirmed that GLUT3 OE significantly increases TMZ/CAPE uptake and cytotoxicity, particularly under fasting conditions. Our findings establish GLUT3 as a multifunctional transporter for TMZ, CAPE, and glucose, thereby enhancing GBM chemosensitivity. These results challenge the prevailing notion that GLUT3’s role in tumors is solely related to glucose transport. Our work suggests tailoring chemotherapy based on GLUT3 expression level in GBM patients and reevaluating GLUT inhibitors in combination with chemotherapeutic agents.

## Introduction

Glioblastoma multiforme (GBM) is the most common and highly aggressive primary malignant brain tumor in adults. Despite advances in surgical techniques, radiation therapy, and chemotherapy, the prognosis for GBM patients remains extremely poor, with a median overall survival of ~15 months and a progression-free survival of only 6 months [1–3]. The standard treatment protocol for GBM includes maximal surgical resection, followed by radiotherapy and concurrent chemotherapy [4]. However, the options for chemotherapeutic agents in treating GBM are extremely limited, with temozolomide (TMZ) being the only first-line drug approved for this indication [5]. Although studies have shown that TMZ can improve survival rates compared to radiotherapy alone, the overall benefit is often modest. Approximately 50% of patients treated with TMZ do not respond to the therapy [6, 7]. Research on TMZ treatment resistance has identified several contributing factors, such as elevated levels of MGMT (O6-methylguanine-DNA methyltransferase), increased activity of DNA repair pathways, the presence of tumor stem cells, and the role of drug efflux transporters [8, 9]. However, these factors do not fully account for all cases of TMZ resistance, suggesting that additional mechanisms may be at play.

TMZ is a small lipophilic molecule, and was initially believed to easily penetrate the blood-brain barrier (BBB) [10]. However, recent findings have challenged this assumption. A study by Zhang’s group demonstrated that inhibiting Wnt signaling could enhance TMZ delivery and sensitize glioma cells to chemotherapy [11], indicating that the entry of TMZ into cells may be a more controlled process than previously thought. Furthermore, some researchers have used model membrane absorption and fluorescence experiments to show that TMZ does not readily penetrate the lipid bilayer and instead binds to the membrane surface with very low affinity [12, 13]. These observations suggest that the uptake of TMA into GBM cells may be facilitated by specific transporter proteins.

GLUT3 (Glucose Transporter 3) is an important glucose transporter that has been extensively studied for its role in tumor proliferation and metastasis. Recent research has shed light on GLUT3’s involvement in chemotherapy resistance. For example, overexpression of GLUT3 has been linked to increased resistance to vincristine in acute myeloid leukemia (AML) cells [14] and to paclitaxel in breast cancer cells [15], likely due to enhanced glucose uptake. Conversely, GLUT3 overexpression has also been shown to enhance sensitivity to arsenite in cervical and triple-negative breast cancers [16] and to gemcitabine in pancreatic cancer [17]. In addition, gene expression profiling demonstrated that GLUT3 levels were significantly elevated in gemcitabine-sensitive non-small cell lung cancer (NSCLC) cell lines and paclitaxel-sensitive MDA-MB-231 cell lines when compared to their drug-resistant counterparts [18, 19]. These findings suggest that GLUT3 has complex and varied roles in tumor chemotherapy, which may differ by tumor type. While glucose transport helps clarify GLUT3’s role in chemoresistance, it does not explain its impact on chemosensitization.

GLUT3 is a member of the GLUT family of transporters, primarily recognized for glucose transport, but emerging evidence indicates that these transporters can also facilitate the transport of other substrates, including urates, dehydroascorbate, and inositol [20]. Clinical oncology applications, such as the use of the radiolabeled glucose analog ^18^F-FDG in diagnostic tumor imaging and the design of glycoconjugates to enhance anti-tumor drug delivery, demonstrate that GLUTs are capable of transporting glucose analogs and glycoconjugates [21, 22]. Recent studies, including our own unpublished work, have shown that GLUT3 can bind to and influence the uptake of chemotherapeutic agents like gemcitabine, enhancing chemotherapy sensitivity of triple-negative breast cancer cells. Given the high expression of GLUT3 in GBM [23] and its potential to transport molecules beyond glucose, it is plausible that GLUT3 could play a role in chemotherapy beyond just glucose transport. This raises the question of whether GLUT3 might also be involved in the transport of other chemotherapy drugs in the context of GBM.

In the present study, we unveiled a novel role of GLUT3 beyond glucose transport in GBM. Our findings demonstrated that GLUT3 can bind and transport both TMZ and CAPE, establishing it as a multifunctional transporter for these chemotherapeutic agents and glucose in GBM. A comprehensive suite of cellular and animal experiments revealed that overexpression of GLUT3 increased the transport of TMZ and CAPE, thereby intensifying the chemosensitivity of GBM. Furthermore, our animal studies highlighted that the combination of GLUT3 overexpression and fasting significantly enhances the efficacy of GBM treatment compared to fasting alone. This discovery offers valuable insights into optimizing clinical chemotherapy regimens for GBM. Overall, our study elucidates the molecular mechanism by which GLUT3 modulates chemosensitivity in GBM through chemotherapeutic drug transport, furnishing a theoretical foundation for intracellular drug transport in GBM and a fresh perspective for its clinical management.

## Results

### GLUT3 expression correlates with glioma malignancy, prognosis, and chemotherapy resistance

**Fig. 1:**
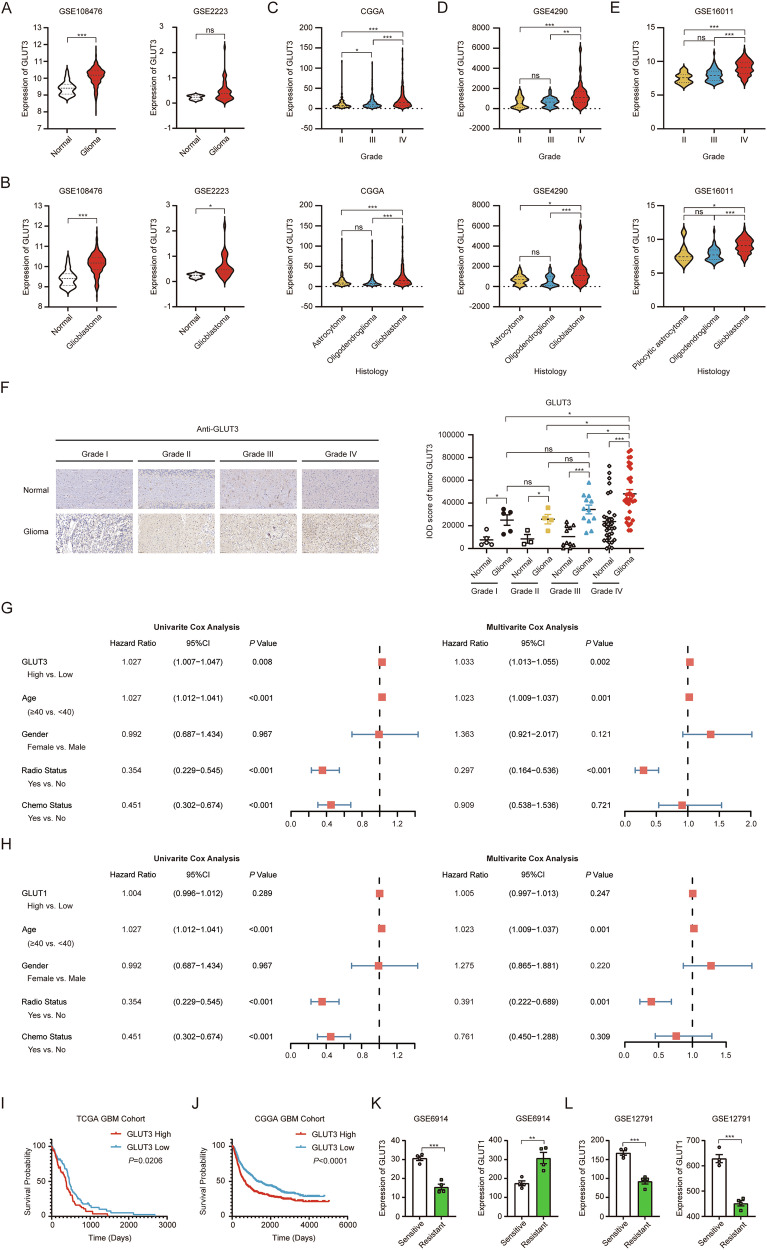
GLUT3 expression correlates with malignancy, prognosis of gliomas, and chemotherapy resistance. **A**, **B** Comparison of GLUT3 mRNA levels between glioma (**A**) /glioblastoma (GBM) **B** and corresponding normal tissues using GSE108476 and GSE2223 datasets. The mRNA levels of GLUT3 were compared in different grades and histology of gliomas using CGGA database (**C**), GSE4290 (**D**) and GSE16011 (**E**) datasets. **F** IHC analysis of GLUT3 in human glioma tissue microarray (TMA). Representative IHC images showing GLUT3 staining patterns in glioma (WHO grade I-IV) versus matched adjacent normal brain tissues (scale bar: 50 μm). Antibody: anti-SLC2A3 (GLUT3, Abcam ab41525, 1:200 dilution). Univariate and multivariate analyses of the association of GLUT3 (**G**) and GLUT1 (**H**) expression and other clinical information with overall survival (OS) in the TCGA GBM cohort. Comparison of overall survival (OS) between GLUT3 high- and low- expressing GBM in the TCGA (**I**) and CGGA (**J**) GBM cohort using Kaplan–Meier plots. Data were analyzed by log-rank test. Expression of GLUT1 and GLUT3 in gemcitabine-sensitive and gemcitabine-resistant NSCLC cells (**K**) and paclitaxel-sensitive and paclitaxel-resistant MDA-MB-231 cells **L** in the GSE6914 dataset and the GSE12791 dataset, respectively. Independent-sample *t*-tests (**F**, **K**, **L**), nonparametric tests (**A**, **B**), or one-way ANOVA with Kruskal-Wallis test (**C**-**E**) were used for statistical analysis by using SPSS 20 (IBM). A *p* value < 0.05 was considered significant (*P < 0.05, **P < 0.01, ***P < 0.001).

To investigate the role of GLUT3 in gliomas, we first analyzed its expression using data from the GSE108476 and GSE2223 datasets. As expected, GLUT3 expression was markedly higher in GBM tissues than in corresponding normal brain tissues across both datasets (Fig. 1A, B). Further analysis revealed a significant increase in GLUT3 expression in WHO grade IV gliomas compared to grades II and III (upper panels of Fig. 1C–E). In addition, GLUT3 expression was elevated in GBM compared to astrocytoma and oligodendroglioma (lower panels of Fig. 1C–E). These findings indicate that the levels of GLUT3 expression rise with increasing glioma malignancy. We validated these findings through immunohistochemical (IHC) analysis of GLUT3 protein expression using tissue microarrays (TMAs) comprising 80 glioma samples across WHO grades I-IV. The IHC results confirmed significant higher GLUT3 expression in grade I-IV gliomas than in corresponding normal brain tissues (Fig. 1F). Quantitative analysis showed no significant differences in GLUT3 expression among grade I-III gliomas, but a notable upregulation was observed in grade IV gliomas compared to lower grades (Fig. 1F), corroborating our prior analyses of GEO and CGGA database (Fig. 1A–E) and solidifying GLUT3’s status as a malignancy-progressive biomarker. Univariate and multivariate Cox regression analyses of clinical variables in the TCGA GBM cohorts identified GLUT3 was an independent prognostic factor for GBM patients (Fig. 1G). In contrast, similar analyses of GLUT1, a major glucose transporter in GBM, revealed no significant impact on patient survival or prognosis in the TCGA GBM cohorts (Fig. 1H). Analysis of GLUT3 expression and overall survival of GBM indicated that patients with high expression of GLUT3 had a lower survival rate compared to those with low expression (Fig. 1I, J). Kaplan–Meier analysis further demonstrated that high GLUT3 expression was associated with lower overall survival rates in GBM patients compared to low expression, suggesting that GLUT3 overexpression is a potential risk factor for GBM.

GLUTs have emerged as promising targets for cancer therapy, with ongoing research into GLUT inhibitors showing their potential as adjuvants in combination with standard chemotherapy. However, the specific role of GLUT3 in chemotherapy remains to be fully elucidated. It remains unclear whether the use of GLUT inhibitors during chemotherapy benefits patients with GBM. To address this, we searched for datasets on tumor chemotherapy resistance and compared the GLUT3 expression between the resistant and sensitive groups. Surprisingly, we found that, in contrast to drug-resistant cells, GLUT3 expression was higher in gemcitabine-sensitive NSCLC cells (Fig. 1K, left panel) and paclitaxel-sensitive MDA-MB-231 cells (Fig. 1L, left panel). This result contradicts the prevailing notion that GLUTs contribute to chemoresistance in tumor cells by facilitating glucose uptake. Since GLUT1 is known to be transcriptionally upregulated in GBM, we further analyzed the association between GLUT1 expression and drug resistance in the above datasets. Unlike GLUT3, GLUT1 expression was significantly increased in gemcitabine-resistant NSCLC cells (right panel of Fig. 1K) and decreased in paclitaxel-resistant MDA-MB-231 cell lines (right panel of Fig. 1L). This suggests that GLUT1 may play different roles in the chemotherapy of different cancer cells. Given the high expression of GLUT3 in GBM and its unexpected association with tumor chemosensitivity, we decided to focus our subsequent studies on the role of GLUT3 in GBM chemosensitivity.

### GLUT3 enhances chemosensitivity of GBM to TMZ and CAPE

**Fig. 2:**
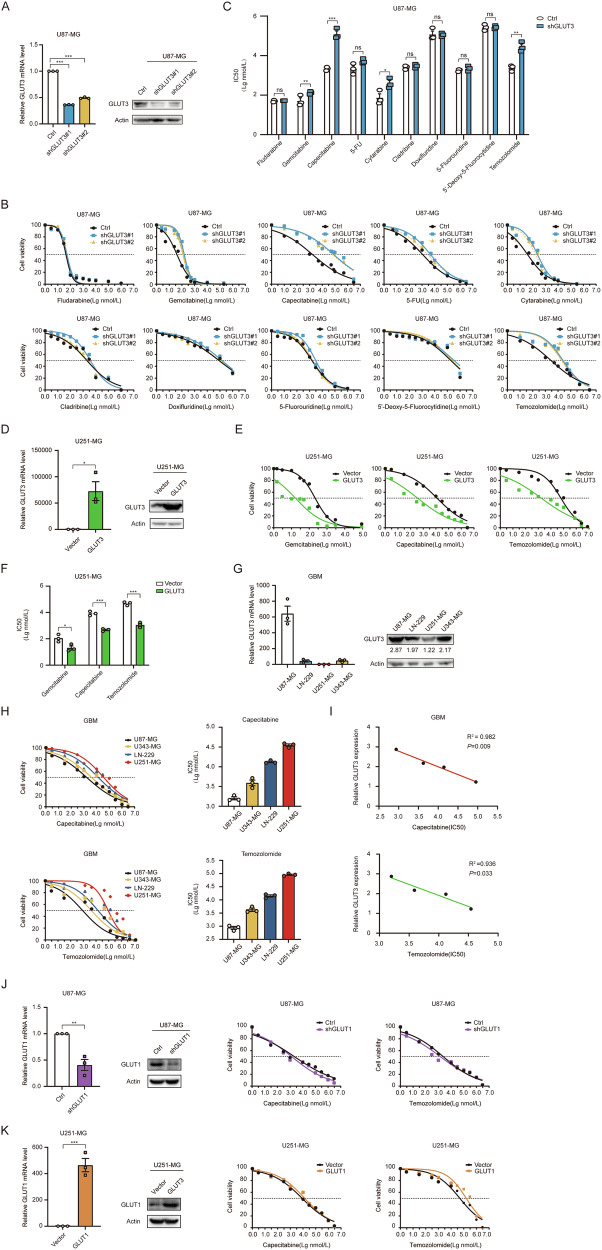
GLUT3 enhances the chemosensitivity of GBM to TMZ and CAPE. **A** GLUT3 KD efficiency in U87-MG cell line. Cell viability (**B**) and IC50 values (**C**) of different nucleoside analog drugs were measured via CCK-8 in U87 cells with or without GLUT3 KD after treatment with various doses of drugs. **D** GLUT3 OE efficiency in U251-MG cell line. Cell viability (**E**) and IC50 values **F** of gemcitabine, capecitabine, and temozolomide were measured via CCK-8 in U251-MG cells with or without GLUT3 OE. **G** GLUT3 mRNA and protein levels in different GBM cell lines. **H** Cell viability and IC50 values of capecitabine (left panels) and temozolomide (right panels) were measured via CCK-8 in different GBM cell lines. **I** The correlation between the relative expression of GLUT3 and its chemotherapy resistance of capecitabine (left panel) and temozolomide (right panel) in different GBM cell lines. **J** Cell viability of capecitabine and temozolomide was measured via CCK-8 in U87 cells with or without GLUT1 KD. **K** Cell viability of capecitabine and temozolomide was measured via CCK-8 in U251-MG cells with or without GLUT1 OE. Data are presented by means ± SEM of (*n* = 3) biologically independent experiments. Independent-sample *t*-tests (**C**, **D**, **F**, **J**, **K**) or one-way ANOVA with Tukey’s post-hoc test **A** were used for statistical analysis by using SPSS 20 (IBM). Pearson’s correlation analysis determined the correlation between two variables (**I**). A *p* value < 0.05 was considered significant (*P < 0.05, **P < 0.01, ***P < 0.001).

We screened nucleoside antitumor agents that might be influenced by GLUT3 in GBM cells, as these agents are typical ribose-containing compounds. The rationale for selecting this class of antitumor drugs was discussed in the previous introductory section. We hypothesized that GLUTs can potentially transport glycosyl compounds other than hexose. To test this hypothesis, we knocked down GLUT3 expression in U87 cells, a GBM cell line with high GLUT3 expression, and confirmed the knockdown efficiency (Fig. 2A). We then investigated the impact of GLUT3 KD on the half-maximal inhibitory concentration (IC50) of nine antitumor nucleoside analogs in U87-MG cells (Fig. 2B, C). TMZ, a first-line clinical chemotherapeutic drug for glioblastoma multiforme (GBM) patients, was included as a positive control (Fig. 2B). The results showed that GLUT3 knockdown significantly increased the IC50 values of various drugs: ~1.6-fold for gemcitabine (GEM), 56.5-fold for capecitabine (CAPE), 4.3-fold for cytarabine, and 10.6-fold for TMZ. This suggests that GLUT3 deficiency decreases the sensitivity of GBM cells to these drugs, particularly to CAPE (Fig. 2C). To further validate this effect, we overexpressed GLUT3 in the U251-MG cell line which has low GLUT3 expression (Fig. 2E), and confirmed the overexpression efficiency (Fig. 2D). The result demonstrated that the IC50 values of GEM, CAPE, and TMZ were reduced by ~81.7%, 94.4%, and 97.7%, respectively, in GLUT3 overexpressing U251-MG cells compared to control cells (Fig. 2E, F). We also assessed the cytotoxic potential of TMZ and CAPE in the normal astrocyte cell line, SVGp12. The dose-response curves of TMZ and CAPE on SVGp12 cells exhibited a classic sigmoidal shape (*R*² > 0.97). Specifically, the CC50 of TMZ was calculated at 103.7 ± 21.1 μM (95% CI: 55.3–174.9 μM) (Supplementary Fig. 2A), while that for CAPE was 163.2 ± 31.8 μM (95% CI: 92.1–265.8 μM) (Supplementary Fig. 2B). These CC50 values indicate that both drugs exhibit low cytotoxicity in this cellular model.

We further confirmed the relationship between GLUT3 expression and GBM chemosensitivity to CAPE and TMZ. We assessed GLUT3 expression in various GBM cell lines and found variability: U87-MG cells exhibited high GLUT3 expression, while the U251-MG cell line demonstrated low GLUT3 expression, and both the U343-MG and LN-229 cells showed moderate expression (Fig. 2G). We determined the IC50 values for these GBM cell lines in response to CAPE and TMZ (Fig. 2H) and used scatter plots to visualize the relationship between GLUT3 expression and GBM chemosensitivity. This revealed a negative correlation between GLUT3 expression and IC50 value of both CAPE (Fig. 2I, upper panel) and TMZ treatment (Fig. 2I, lower panel). Given the upregulation of GLUT1 in GBM, we also investigated the effects of GLUT1-KD and OE on the chemosensitivity of U87-MG (Fig. 2J) and U251-MG (Fig. 2K) cell lines. The efficiency of GLUT1 KD and GLUT1 OE was confirmed (left panels of Fig. 2J, K). The results showed that GLUT1 does not significantly affect GBM chemotherapy sensitivity (right panels of Fig. 2J, K). This suggests that GLUT3’s role in modulating GBM chemosensitivity to TMZ and CAPE may be specific to GLUT3, rather than a general characteristic of GLUTs.

### The diverse roles of GLUT3 in GBM cells with or without TMZ/CAPE treatment

**Fig. 3:**
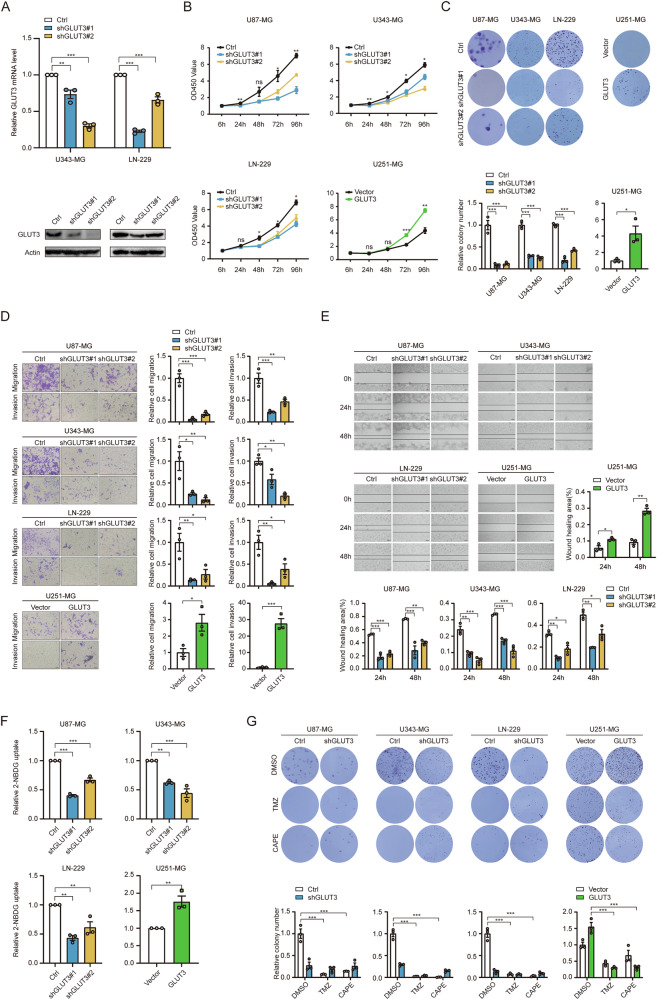
The effect of GLUT3 on GBM cells varies significantly in conditions with or without TMZ/CAPE treatment. **A** GLUT3 KD efficiencies in U343-MG and LN-229 cell line. CCK8 (**B**) and colony formation **C** assays were performed to determine the cell proliferation ability of U87-MG cells, U343-MG cells, and LN-229 cells with or without GLUT3 KD and U251-MG cells with or without GLUT3 OE. **D** Transwell assay for invasion and migration was performed on U87-MG cells, U343-MG cells, and LN-229 cells with or without GLUT3 KD, and U251-MG cells with or without GLUT3 OE. **E** Wound healing assay was performed to detect the cell migration ability of U87-MG cells, U343-MG cells, and LN-229 cells with or without GLUT3 KD and U251-MG cells with or without GLUT3 OE. The wound closure percentage was calculated by Image J software. **F** 2-NBDG uptake by GLUT3 KD U87-MG, U343-MG, and LN-229 cells, as well as 2-NBDG uptake by GLUT3 OE U251-MG cells relative to control (Ctrl) cells. Cells were cultured with fluorescent probe 2-NBDG (100 μM) in glucose-free DMEM for 30 min at 37 °C; fluorescence signals were detected and normalized by cell number. **G** Colony formation assay was performed to determine cell proliferation ability. U87-MG, U343-MG, and LN-229 cells with or without GLUT3 KD were treated with DMSO or TMZ (200 μM) or CAPE (400 μM) once every two days until macroscopic clones were formed. Meanwhile, U251-MG cells with or without GLUT3 OE were manipulated as above. Clones were calculated by Image J software. Data are presented by means ± SEM of (*n* = 3) biologically independent experiments. One-way ANOVA with Tukey’s post-hoc test (**A**, **B**–**F**, U87-MG, U343-MG, LN-229 cell line, and **G**) or independent-sample *t*-tests (**B**–**F**, U251-MG cell line) were used for statistical analysis by using SPSS 20 (IBM). A *p*-value < 0.05 was considered significant (*P < 0.05, **P < 0.01, ***P < 0.001).

Further exploration into the distinct roles of GLUT3 revealed significant variations in its influence on the phenotype of GBM cells and their response to TMZ/CAPE treatment at the cellular level. The efficiency of GLUT3 KD in U343-MG and LN-229 cell lines is shown in Fig. 3A. Using CCK8 and colony formation assays, we found that loss of GLUT3 inhibited proliferation in U87-MG, U343-MG, and LN-229 cells, while GLUT3 OE enhanced this ability in U251-MG cells (Fig. 3B, C). Transwell and wound healing assays demonstrated that GLUT3 deficiency reduced migration and invasion in U87-MG, U343-MG, and LN-229 cells, while GLUT3 OE notably enhanced these capabilities in U251 cells (Fig. 3D, E). Fluorescent glucose analog 2-NBDG uptake assays indicated that GLUT3 deficiency reduced glucose uptake in U87-MG, U343-MG, and LN-229 cells, whereas GLUT3 overexpression increased uptake in U251-MG cells (Fig. 3F). These findings confirm that GLUT3 promotes GBM proliferation and metastasis in the absence of chemotherapeutic drugs, consistent with its role in glucose uptake.

Subsequently, we investigated the impact of GLUT3 KD or OE on chemotherapeutic sensitivity of GBM cells. Colony formation assays revealed that GLUT3 deficiency mitigated the TMZ/CAPE-induced reduction in colony formation in U87-MG, U343-MG, and LN-229 cells. Conversely, GLUT3 OE amplified the TMZ/CAPE-induced decrease in colony numbers (Fig. 3G). This indicates that high levels of GLUT3 enhance TMZ/CAPE cytotoxicity in GBM cells, while GLUT3 KD has the opposite effect. This contrasts with GLUT3’s role in promoting GBM cell proliferation in absence of these chemotherapeutic agents. Thus, GLUT3 may have additional roles beyond glucose transport during TMZ/CAPE treatment.

### GLUT3 promotes the uptake of extracellular temozolomide and capecitabine

**Fig. 4:**
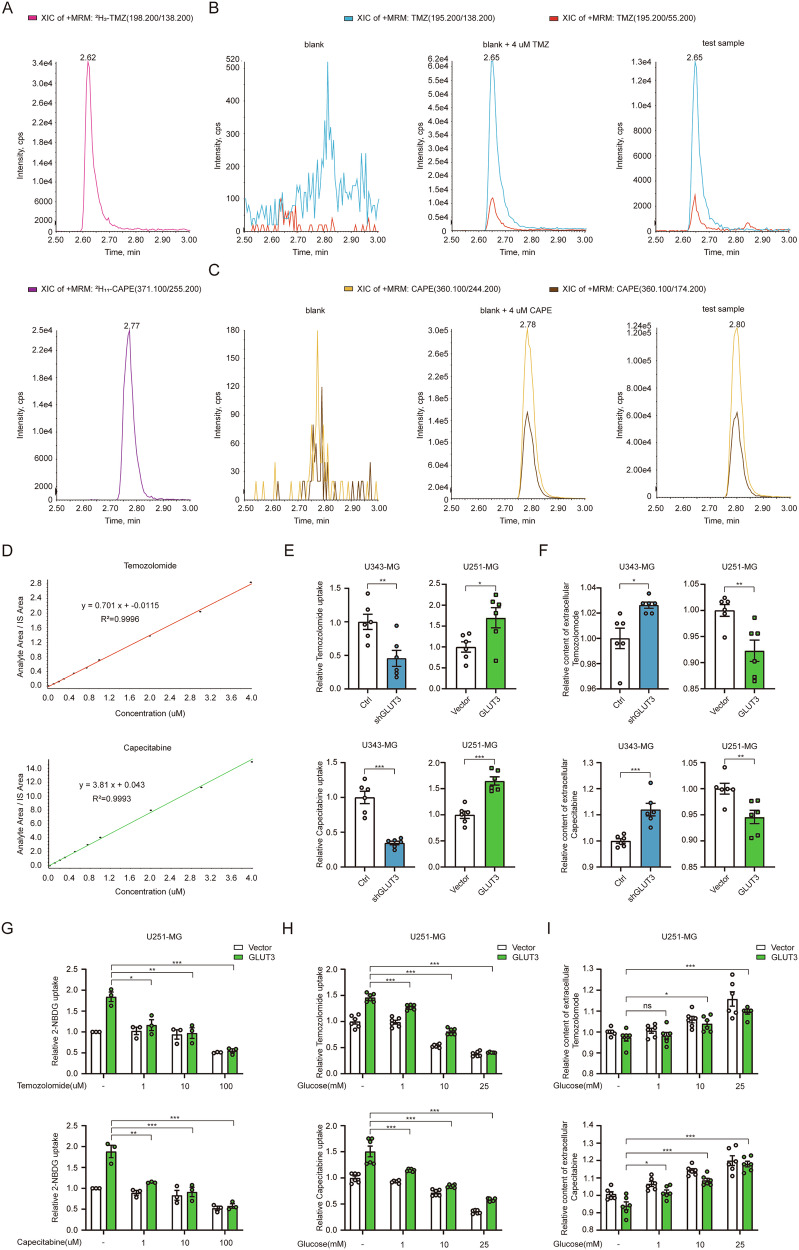
GLUT3 promotes the uptake of extracellular temozolomide and capecitabine. **A** Representative chromatograms of IS (internal standard). IS is added to each sample as a mixture of ^2^H_3_-Temozolomide (upper panel) and ^2^H_11_-Capecitabine (lower panel). The sample’s final concentrations of ^2^H_3_-Temozolomide and ^2^H_11_-Capecitabine were 60 ng/ml and 12 ng/ml, respectively. **B** Representative chromatograms of TMZ in blank (left panel), blank with TMZ (middle panel, the final concentration of TMZ was 0.64 μM), and test sample (right panel). Cells were treated with drug-free transport buffer (blank), transport buffer containing 200 μM TMZ (test sample) at 37 °C for 15 min. The harvested cells were extracted with 80% methanol and prepared for LC-MS/MS analysis. **C** Representative chromatograms of CAPE in blank (left panel), blank with CAPE (middle panel, the final concentration of CAPE was 0.64 μM), and test sample of CAPE (right panel). Cells were treated with drug-free transport buffer (blank), transport buffer containing 200 μM CAPE (test sample) at 37 °C for 15 min. **D** Calibration curves for TMZ (upper panel) and CAPE (lower panel). **E** The uptake of TMZ (upper panel) and CAPE (lower panel) by GLUT3 KD (U343-MG) or GLUT3 OE (U251-MG) cells. Cells were treated with transport buffer or transport buffer containing 200 μM TMZ or CAPE at 37 °C for 15 min. The cell lysates were used for LC-MS/MS for intracellular TMZ and CAPE levels. **F** The levels of remaining extracellular TMZ (upper panel) and CAPE (lower panel) after uptake by GLUT3 KD (U343-MG) or GLUT3 OE (U251-MG) cells. **G** Inhibition of glucose uptake of TMZ or CAPE. 2-NBDG fluorescence assays were performed in GLUT3-OE vs. vector control U251-MG cells with TMZ (1, 10, 100 μM) or CAPE (1, 10, 100 μM) for 24 h. **H** Glucose concentration-dependent inhibition of chemotherapeutic drug uptake. U251-MG cells (GLUT3-OE and control vector) were exposed to TMZ (upper panel) and CAPE (lower panel) in presence of varying glucose concentration (1, 10, 25 mM). **I** Extracellular drug accumulation quantified by LC-MS/MS: Upper panel, TMZ levels under glucose modulation; Lower panel, CAPE levels under glucose modulation. Data are presented as mean ± SEM (*n* ≥ 5 biological replicates). Statistical analysis was performed using two-tailed unpaired *t*-tests **E**, **F**, or one-way ANOVA with Tukey’s post-hoc test (**G**–**I**) using SPSS 20 (IBM). Significance thresholds: *P < 0.05, **P < 0.01, ***P < 0.001.

To investigate whether GLUT3 enhances the cytotoxic effect of TMZ and CAPE by regulating their transport, we assessed the impact of GLUT3 KD and OE on intracellular and extracellular levels of these drugs using LC-MS/MS. The analysis employed isotopically labeled standards (^2^H_3_-Temozolomide and ^2^H_11_-Capecitabine) as internal standards (IS) and identified characteristic ion fragmentation peaks. Representative chromatograms of IS are shown in Fig. 4A. Blank samples (cells incubated with transport buffer) displayed minimal baseline fluctuations near the retention times of TMZ or CAPE (left panels of Fig. 4B, C), while the middle panels show the chromatograms for TMZ and CAPE standards used for calibration. The determination of TMZ and CAPE peaks relied on specific transitions in positive ion mode: 195.2 > 138.2 (quantifier) and 195.2 > 55.2 (qualifier) for TMZ, and 360.1 > 244.2 transition (quantifier) and 360.1 > 174.2 (qualifier) for CAPE. Representative chromatograms of TMZ and CAPE in test samples (cells incubated with 200 μM TMZ or CAPE) are shown in the right panels of Fig. 4B, C, exhibiting excellent signal-to-noise ratios compared to blanks.

Calibration curves for TMZ and CAPE were generated via linear regression analysis, plotting the peak area ratio (analyte/IS) against the concentration (Fig. 4D). The curves showed strong linear correlations (*R*² = 0.9996 for TMZ and *R*² = 0.9993 for CAPE), enabling accurate determination of drug concentrations in samples. Results indicated that GLUT3 KD in U343-MG cells reduced TMZ uptake by 54.3% and CAPE uptake by 65.5% (left panel of Fig. 4E), while GLUT3 OE in U251-MG cells increased uptake of both drugs by ~70% (right panel of Fig. 4E). Extracellular drug levels were also assessed. In GLUT3-KD U343-MG cells, extracellular TMZ and CAPE were 3% and 12% higher than controls (left panel of Fig. 4F), whereas in GLUT3 OE U251-MG cells, extracellular concentrations decreased by 7.7% and 5.4%, respectively (right panel of Fig. 4F). The less pronounced changes in extracellular levels, compared to intracellular levels (Supplementary Fig. 1), may stem from higher extracellular drug concentrations and shorter incubation time. Nonetheless, the trend in extracellular levels aligns with the intracellular uptake changes, supporting GLUT3’s role in facilitating TMZ and CAPE uptake in GBM cells.

We further investigated the interaction between GLUT3’s promotion of TMZ and CAPE uptake and its glucose uptake function. Using 2-NBDG uptake assays in GLUT3-OE U251-MG and control cells, we found that in control cells, glucose uptake was minimally affected by TMZ until concentration reached 100 μM. In contrast, GLUT3 OE cells showed a 36.8%, 47.2%, and 70.1% reduction in 2-NBDG uptake at 1, 10, 100 μM TMZ, respectively (upper panel of Fig. 4G). Similar results were observed for CAPE, with GLUT3 OE cells exhibiting 39.4%, 51.5%, and 69.3% reductions in 2-NBDG uptake at 1, 10, and 100 μM CAPE (lower panel of Fig. 4G). These findings suggest that GLUT3 overexpression increases GBM cell sensitivity to low concentrations of TMZ and CAPE, indicating that these compounds affect glucose transport through GLUT3. Additionally, we examined how glucose levels influence TMZ and CAPE uptake. As glucose concentration increased, TMZ and CAPE uptake decreased in both control and GLUT3-OE cells. However, GLUT3-OE cells demonstrated greater sensitivity to low glucose (1 mM) than controls (Fig. 4H). Changes in extracellular TMZ and CAPE levels aligned with uptake assay results (Fig. 4I). Based on our prior analysis of GLUT3’s potential to non-glucose small molecules, along with our findings on TMZ and CAPE’s inhibition of GLUT3-mediated glucose transport and glucose influence on TMZ/CAPE transport, we hypothesize that GLUT3 directly binds and transports TMZ and CAPE in GBM cells.

### GLUT3 is predicted to directly bind TMZ and CAPE

**Fig. 5:**
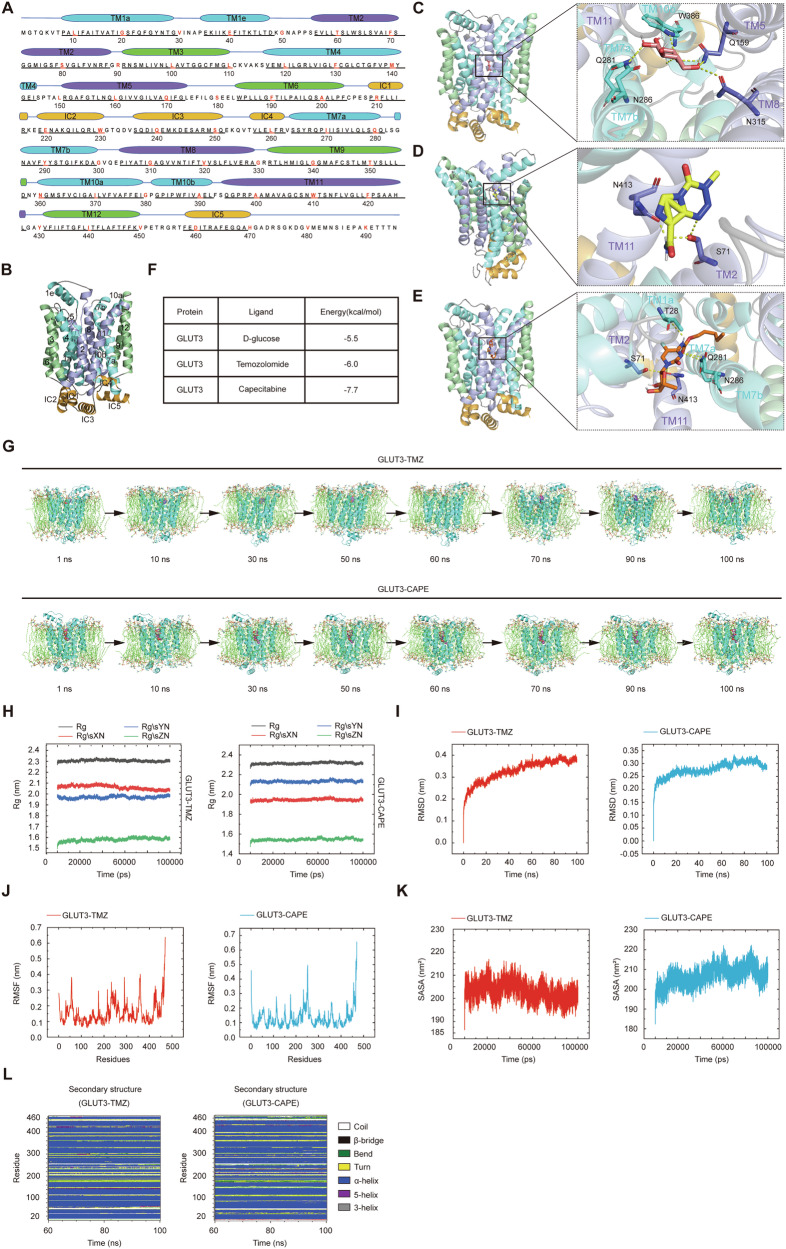
GLUT3 is predicted to bind TMZ and CAPE directly. The amino acid sequence with the assignment of secondary structures (**A**) and the three-dimensional structure of GLUT3 (**B**). The transmembrane region of GLUT3 contains 12 transmembrane segments (TMs) folded into the N-terminal and C-terminal domains, each consisting of two 3-TM repeats. IC1–4 are helices connecting the N-terminal and C-terminal domains. IC1-4 and the C-terminal helix IC5 constitute the intracellular helical (ICH) domain. The corresponding TMs of the four 3-TM repeats are colored the same. In this way, the same blue-violet-green color scheme was used for each 3-TM repeat sequence. The overall docking structure of GLUT3 with D-glucose (**C**), TMZ (**D**), or CAPE (**E**) was simulated using AutoDock Vina software. **F** The binding energy of the docking model of GLUT3 with glucose/TMZ/CAPE. **G** Representative snapshots of molecular dynamics (MD) simulation of GLUT3 with TMZ (upper panel) or CAPE (lower panel) at different time points. **H** Rg values of GLUT3 in GLUT3-TMZ (left panel) and GLUT3-CAPE (right panel) complexes during the 100 ns simulations. **I** RMSD of GLUT3-TMZ (left panel) and GLUT3-CAPE (right panel) complexes obtained from 100 ns MD simulation. The RMSD of GLUT3-TMZ and GLUT3-CAPE is shown in red and blue, respectively. **J** RMSF of GLUT3 amino acid residues in GLUT3-TMZ (left panel) and GLUT3-CAPE (right panel) complexes, respectively. The RMSF of GLUT3-TMZ and GLUT3-CAPE are shown in red and blue, respectively. **K** SASA of GLUT3-TMZ (left panel) and GLUT3-CAPE (right panel) complex. The SASA of GLUT3-TMZ and GLUT3-CAPE are shown in red and blue, respectively. **L** The secondary structures analysis of GLUT3 in complex with TMZ (left panel) and CAPE (right panel), respectively. White for Coil, black for β-bridge, green for Bend, yellow for Turn, dark blue for α-Helix, purple for 5-Helix, and grey for 3-Helix. All structure figures were prepared with PyMOL.

To test the hypothesis that GLUT3 can directly bind to TMZ or CAPE, we performed molecular docking simulations using AutoDock vina with GLUT3 (PDB code: 4ZW9). The GLUT3 structure features a major facilitator superfamily (MFS) fold with 12 transmembrane segments (TMs) in the N- and C-terminal domains, connected by intracellular helices (IC1-5) (Fig. 5A, B). Yan’s group previously reported that D-glucose binds asymmetrically within GLUT3’s central cavity, with hydrogen bonds formed primarily with residues from the C-terminal domain. Our molecular docking results confirmed these interactions, showing that GLUT3 residues Q159, Q281, N286, N315, and W386 contribute to D-glucose binding (Fig. 5C).

In our docking model of the GLUT3-TMZ complex, TMZ forms two hydrogen bonds with S71 (TM2) and N413 (TM11) of GLUT3 (Fig. 5D). Additional van der Waals and hydrophobic interactions, primarily from the N-terminus, stabilize the binding. Due to its larger and more complex structure, TMZ’s binding differs significantly from glucose. The GLUT3-CAPE complex reveals CAPE forming hydrogen bonds with N286, T28, S71, N413, and Q281 (Fig. 5E). The interacting amino acids partially overlap with those in GLUT3-TMZ and GLUT3-glucose complexes, likely due to CAPE’s structural similarities to glucose and TMZ components. Notably, the binding energy of GLUT3 to TMZ and CAPE was lower than to glucose (Fig. 5F), suggesting a higher affinity. This aligns with our substrate uptake competition assays, where lower concentrations of TMZ and CAPE were needed to inhibit glucose transport compared to the reverse (Fig. 4G–I).

To further validate these interactions, we conducted 100 ns molecular dynamics (MD) simulations, saving trajectory data every 2 ps. Visualizations using PyMOL showed that the CAPE’s binding to GLUT3 remained relatively stable, while TMZ’s orientation and position varied more over time (Fig. 5G). Structural parameters, including the radius of gyration (Rg), root mean square deviation (RMSD), root mean square fluctuation (RMSF), solvent-accessible surface area (SASA), and secondary structure analysis, confirmed the stability of both complexes. In the GLUT3-TMZ system, Rg value showed slight variations, while GLUT3-CAPE’s Rg remained constant (Fig. 5H). RMSD values for both the complexes stabilized after 60 ns (Fig. 5I), and RMSF analysis revealed greater fluctuations in the intracellular helical domains (Fig. 5J). SASA analysis showed stabilized fluctuations after 60 ns, with CAPE’s SASA slightly higher than TMZ’s (Fig. 5K). Secondary structures analysis confirmed α-Helix as the dominant secondary structure, with notable transformation observed in the IC region and TM2 during the late period of simulation (Fig. 5L). These MD results corroborated the docking simulations, providing a solid theoretical foundation for GLUT3’s direct binding to TMZ and CAPE.

### GLUT3 functions as a multifunctional transporter for TMZ, CAPE, and glucose

**Fig. 6:**
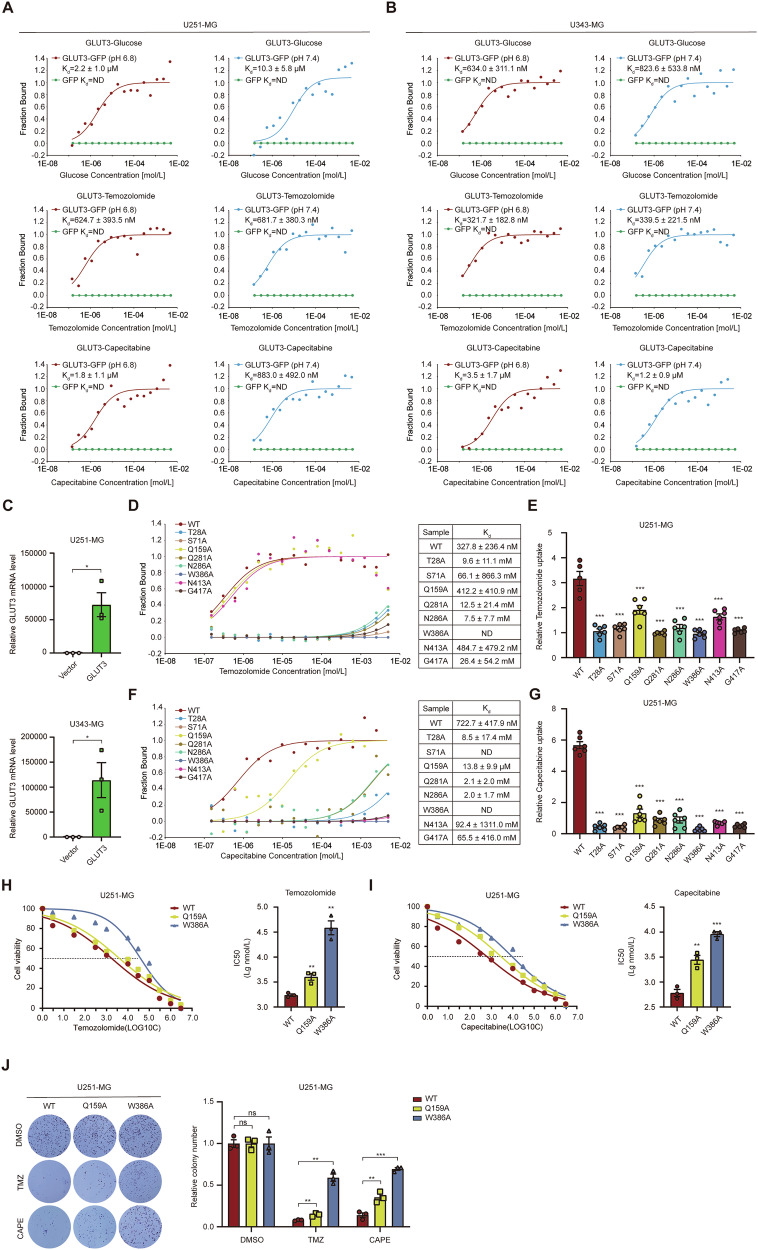
GLUT3 is a TMZ-CAPE-glucose multifunctional transporter. **A,** **B** Binding of GLUT3 with glucose (upper panels), TMZ (middle panels), or CAPE (lower panels) detected by MST assay. Cell lysates used in the MST assay were obtained from U251-MG (**A**) and U343-MG (**B**) cells stably expressing GLUT3-GFP and corresponding control, and the assays were performed at pH 6.8 or 7.4, respectively. Binding curves and *K*_d_ (dissociation constant) values are also shown. **C** GFP-GLUT3 OE efficiency in U251-MG (upper panel) and U343-MG (lower panel) cell lines. **D** Detection of TMZ binding to GLUT3 WT or its mutants by MST assay. **E** Comparison of TMZ uptake by U251-MG cells stably expressing GLUT3 mutants and WT GLUT3. **F** Detection of CAPE binding to GLUT3-GFP WT or its mutants by MST assay. **G** Comparison of CAPE uptake by U251-MG cells stably expressing GLUT3 mutants and WT GLUT3. Effect of GLUT3 mutations on IC50 values of TMZ (**H**) and CAPE (**I**) in U251-MG cells detected by CCK-8 assay. **J** Effect of GLUT3 mutations on colony formation of U251-MG cells. Cells overexpressed with GLUT3 Q159A, W386A, or WT were treated with DMSO, TMZ (200 μM), or CAPE (400 μM) once every two days until macroscopic clones were formed. Clones were calculated by Image J software. Data are presented by means ± SEM of (*n* = 3) (**A**–**D**, **F**, **H**–**J**) or (*n* = 6) **E**, **G** biologically independent experiments. Independent-sample *t*-tests (**C**, **H**–**J**) or one-way ANOVA with Tukey’s post-hoc test **E**, **G** were used for statistical analysis by using SPSS 20 (IBM). A *p*-value < 0.05 was considered significant (*P < 0.05, **P < 0.01, ***P < 0.001).

To confirm GLUT3’s binding to TMZ and CAPE, we employed microscale thermophoresis (MST) to measure interactions between GLUT3 and these ligands, comparing them to GLUT3’s interactions with glucose. MST quantifies biomolecular interactions by detecting fluorescence changes in response to temperature variations. We constructed a GLUT3-GFP fusion protein and performed MST assays in U343-MG and U251-MG cell lysates. The results showed that GLUT3-GFP’s binding to glucose increased with glucose concentration until saturation (upper panels of Fig. 6A, B), with dissociation constants (*K*_d_) in the micromolar range (below 1 μM in U343-MG cells and 2–10 μM in U251-MG cells). This aligns with Singh’s findings of a *K*_d_ value of 0.28 μM for glucose binding to GLUT3 in rat brains [24]. Our studies on breast cancer cell lines indicated that the binding between GLUT3 and its substrates was influenced by pH. However, in GBM cell lines, pH showed no significant effect on glucose binding (Fig. 6A, B). The MST curves for GLUT3’s binding to TMZ and CAPE mirrored those of glucose, with *K*_d_ values of ~0.3–0.6 μM for TMZ and 1 to 3 μM for CAPE (middle and lower panels of Fig. 6A, B). No binding was observed for GFP, confirming the specificity of GLUT3-ligand interactions. The efficiency of GLUT3 overexpression in U343-MG and U251-MG cells is confirmed in Fig. 6C.

To further validate these bindings, we conducted site-directed mutagenesis on eight key amino acids involved in hydrogen bonding. Mutants T28A, S71A, Q281A, N286A, W386A, and G417A showed significantly reduced affinity for TMZ (*K*_d_ increased > 1000-fold) and CAPE (*K*_d_ increased > 1000-fold for seven mutants), leading to 60–70% reduction in TMZ uptake and 75–95% reduction in CAPE uptake (Fig. 6D–G). Notably, while N413A mutant (Fig. 6D) did not affect TMZ binding, it reduced TMZ uptake by 40–50%, suggesting a role in transporter conformational changes. Q159A also reduced uptake without affecting binding. These mutagenesis studies further confirm the crucial role of these amino acids in the binding and transport of TMZ and CAPE by GLUT3.

We selected Q159A and W386A mutants for functional studies due to their distinct impact on GLUT3 function. Q159A causes moderate disruption of GLUT3-mediated TMZ and CAPE transport without affecting binding, while W386A severely impairs both binding and transport (Fig. 6D-G). We prioritized these mutants to investigate how varying degrees of GLUT3 dysfunction change GBM cells’ response to chemotherapy. Using the CCK-8 assay, we assessed their impact on TMZ (Fig. 6H) and CAPE (Fig. 6I) chemosensitivity in U251-MG cells. W386A, a loss-of-function mutant, significantly raised the IC50 values of both drugs in GBM cells (TMZ: 21.3-fold increase in IC50, *P* = 0.001; CAPE: 21.3-fold increase in IC50, *P* < 0.001) (Fig. 6H, I), consistent with its compromised binding and transport abilities. Q159A also increased the IC50 values, especially for CAPE (TMZ: 1.3-fold increase in IC50, *P* = 0.007; CAPE: 3.6-fold increase in IC50, *P* = 0.004) (Fig. 6H, I). Colony formation assays confirmed that under TMZ and CAPE treatment, both mutants showed increased colony numbers compared to the GLUT3 WT group (Fig. 6J). Under these treatment conditions, the number of colonies in the Q159A (TMZ: 0.97-fold increase in colony numbers, *P* = 0.005; CAPE: 1.7-fold increase in colony numbers, *P* = 0.008) and W386A (TMZ: 7.3-fold increase in colony numbers, *P* = 0.001; CAPE: 4.4-fold increase in colony numbers, *P* < 0.001) mutant groups increased considerably compared to the GLUT3 WT overexpression group (Fig. 6J), indicating reduced chemosensitivity. Notably, the W389A mutants exhibited a more pronounced increase than the Q159A mutant. Overall, these results demonstrate that the impact of these two mutants on the chemosensitivity of glioma to TMZ and CAPE correlates with the degree of alteration in their binding and transport functions, highlighting GLUT3’s role in enhancing GBM chemosensitivity by direct binding and transport of these drugs.

### Fasting potentiates the chemosensitivity of GLUT3-overexpressing GBM to TMZ and CAPE in vivo

**Fig. 7:**
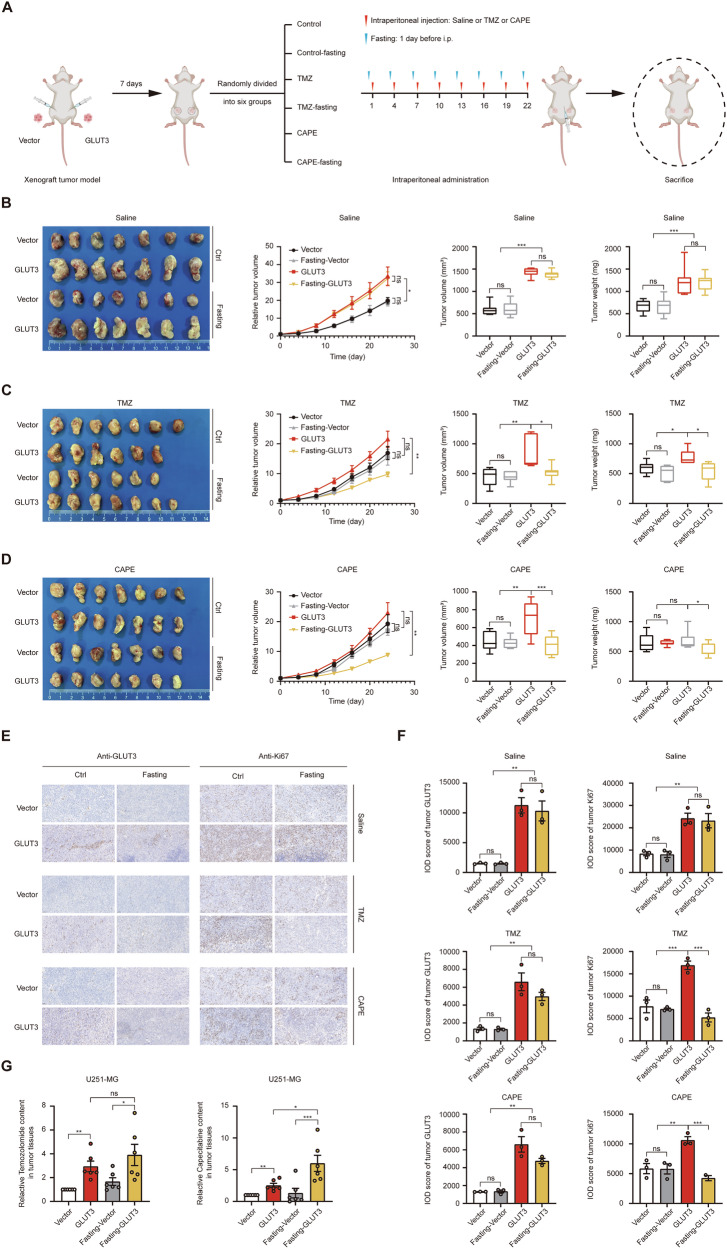
Fasting further enhances the chemosensitivity of GLUT3 overexpressing GBM to TMZ and CAPE in vivo. **A** Scheme of GBM xenograft model establishment and group dosing. 1 × 10^6^ U251-MG cells stably transfected with empty vector or GLUT3 overexpression vector were injected into the left or right axillary fossae of 4-week-old nude mice, respectively. Seven days after tumor injection, when tumor formation is observable, the mice were randomly divided into 6 groups, including control (saline), TMZ-treated (temozolomide 40 mg/kg/dose), CAPE-treated groups (capecitabine 20 mg/kg/dose), and their respective fasting co-treated groups. Intravenous administration was then started every three days, and the combined fasting group was required to undergo a 24 h fast before administration. **B** The effect of GLUT3 overexpression combined with fasting on tumor growth. **C** The effect of GLUT3 overexpression combined with fasting on tumor growth under TMZ treatment. **D** The effect of GLUT3 overexpression combined with fasting on tumor growth under CAPE treatment. The tumor volume is generally calculated by the formula *V* = (length*width^2^)/2. IHC staining (**E**) and score (**F**) of GLUT3 and Ki67 of the tumor samples from the above groups. **G** Relative content of temozolomide (left panel) and capecitabine (right panel) in tumor tissues with GLUT3 overexpression and the combination of GLUT3 overexpression and fasting. Data are presented by means ± SEM of (*n* = 3) **F** or (*n* = 6) **C** or (*n* = 7) **B**–**D** biologically independent experiments. Independent-sample *t*-tests (**B**–**D** second panels), paired *t*-tests (**G**), or one-way ANOVA with Tukey’s post-hoc test (**B**–**D** third and fourth panels and **F**) were used for statistical analysis by using SPSS 20 (IBM). A *p*-value < 0.05 was considered significant (*P < 0.05, **P < 0.01, ***P < 0.001).

Given that glucose may compete with TMZ and CAPE for GLUT3 binding, low-glucose conditions could favor drug binding to GLUT3. We hypothesized that low glucose, coupled with high GLUT3 expression, would enhance GBM chemosensitivity to these drugs. To test this, we established a U251-MG cell-derived xenograft model in nude mice (Fig. 7A). U251-MG cells harboring either a negative vector or a GLUT3-overexpressing vector were injected into the axillary fossae of four-week-old nude mice. After tumor formation, mice were divided into six groups: control, TMZ-treated, CAPE-treated, and corresponding fasting groups. Control, TMZ, and CAPE groups received a standard diet, while fasting groups fasted for 24 h before each saline or drug administration. Tumor diameters were measured every four days, and mice were euthanized when tumors reached ~1.5 cm^3^. Tumor growth curves, final volumes, and weights were compared.

In saline-treated groups, GLUT3 overexpression significantly accelerated GBM growth, with fasting having no effect in either GLUT3-overexpressing or empty vector groups (Fig. 7B). As anticipated, TMZ and CAPE treatment slowed than in the saline groups, reducing tumor weights and volumes (Fig. 7C, D). Notably, fasting significantly inhibited tumor growth in GLUT3-overexpressing mice treated with TMZ or CAPE compared to non-fasting counterparts (Fig. 7C, D). Immunohistochemical staining confirmed GLUT3 overexpression in tumor tissues (left panels of Fig. 7E, F), and Ki67 staining indicated fasting enhanced tumor growth inhibition in GLUT3-overexpressing mice during chemotherapy (right panels of Fig. 7E, F). This suggests that fasting and GLUT3 synergistically enhance TMZ and CAPE cytotoxicity on GBM cells, potentially via altered blood glucose levels. To validate the effect of fasting on glycemia, we performed comparative measurements of blood glucose concentrations in ad libitum-fed versus 24 h fasted mice. Fed mice exhibit much higher blood glucose levels than fasted mice (Supplementary Fig. 3). In fast mice, reduced glucose competition may increase opportunities for TMZ and CAPE to bind to GLUT3, enhancing drug uptake and chemosensitivity.

Using LC-MS/MS, we measured TMZ and CAPE levels in tumor tissues. In GLUT3 overexpressing tumors, TMZ and CAPE concentrations were ~1.95-fold and 1.52-fold higher than controls (Fig. 7G), confirming that GLUT3 overexpression enhances drug uptake. Compared to the saline groups, TMZ and CAPE treatment attenuated GLUT3’s pro-proliferative effect under normal dietary conditions. In fasting groups, TMZ and CAPE levels in GLUT3-overexpressing tumors were 1.34-fold and 3.54-fold higher than controls (Fig. 7G), further supporting that fasting enhances GLUT3-mediated drug uptake, particularly for CAPE. Although TMZ significantly inhibited tumor growth in fasting GLUT3-overexpressing mice (Fig. 7C), the increase in tumor TMZ levels was not statistically significant compared to the regular diet group (left panel of Fig. 7G), possibly due to rapid TMZ metabolism and the timing of measurement. These in vivo experimental results confirm that GLUT3 enhances GBM chemosensitivity through transporting TMZ and CAPE, and that fasting amplifies this effect. These findings advance our understanding of GLUT3’s molecular mechanism and hold significant promise for optimizing GBM chemotherapeutic strategies.

## Discussion

Our study demonstrates that overexpressing GLUT3 increases the sensitivity of GBM cells to TMZ and CAPE, whereas GLUT3 k’s knockdown reduces this chemosensitivity. This contradicts GLUT3 tumor-growth-promoting role observed under non-chemotherapeutic conditions. Importantly, this enhanced chemosensitivity does not stem from GLUT3’s glucose transport function. Our research first reveals that GLUT3 elevates GBM cells’ chemosensitivity to TMZ and CAPE through active transport of these compounds. We confirmed GLUT3’s multifunctional transport activity for TMZ, CAPE, and glucose through substrate binding/uptake assays, molecular docking, dynamics simulations, and mutagenesis studies. The *K*_d_ values for TMZ and CAPE binding to GLUT3 are ~0.3–0.7 μM and 0.9–3.5 μM, respectively, in the tested GBM cell lines, while the glucose binding *K*_d_ values range from 1 to 10 μM. Using a GBM cell-derived xenograft (CDX) mouse model, we further validated that GLUT3 overexpression boosts tumor uptake of TMZ and CAPE, enhancing drug sensitivity. These findings challenge the view that GLUT3’s chemotherapeutic role is solely linked to glucose transport, suggesting a reevaluation of GLUT inhibitors’ use in tumor therapy, especially when combined with chemotherapeutic agents. Our study offers new insights into enhancing GBM chemotherapy sensitivity and highlights the potential limitation of GLUT inhibitors in cancer treatment.

TMZ, chemically known as 3,4-dihydro-3-methyl-4-oxoimidazo[5,1-d]azepine-2-carboxamide, is a DNA alkylating agent first identified for its anti-tumor properties in the 1940s [9]. It received European approval for recurrent glioblastoma treatment in 1999 and was later approved by the FDA for newly diagnosed glioblastomas in 2005 [25]. This orally administered drug effectively crosses the blood-brain barrier, making it particularly suitable for brain tumor treatment [26]. TMZ exerts its cytotoxic effect by methylating guanine residues in DNA, leading to O^6^-methylguanine formation, DNA mismatches, and apoptosis [25, 27]. However, the enzyme methylguanine-DNA methyltransferase (MGMT) can repair this damage by transferring methyl groups from the O^6^ site of guanine to cysteine residues, reducing the effectiveness of alkylating agents and potentially causing chemoresistance. Methylation of the MGMT gene promoter silences the gene, reduces DNA repair capacity, and increases TMZ sensitivity. While alterations in DNA repair mechanisms, especially MGMT promoter methylation, are key factors in TMZ resistance in GBM [9, 28], combining MGMT inhibitors with TMZ has been challenging due to toxicity, drug interactions, and the presence of additional resistance mechanisms that may affect treatment outcomes [29–32]. Identifying safer TMZ sensitizing strategies is a critical unmet clinical need [33, 34]. Drug efflux is another important mechanism of TMZ resistance. Overexpression of efflux transporters like P-glycoprotein and BCRP can decrease intracellular TMZ levels, promoting resistance [9, 35]. High expression levels of these transporters correlated with poor prognosis and lower survival rates in TMZ-treated patient [36], underscoring their role in resistance. However, the mechanism of TMZ cellular uptake remains unclear. Our study is the first to show that GLUT3 facilitates TMZ transport into GBM cells, enhancing chemosensitivity. This novel finding may inform patient selection and improve TMZ treatment strategies in GBM.

TMZ’s chemical structure resembles that of uric acid, a known substrate for GLUT9 and GLUT12, as both feature a fused ring system with an imidazole ring and a six-membered ring containing nitrogen atoms. These structure similarities imply potential overlaps in their binding and transport characteristics. GLUT9, which prefers urate over glucose, and GLUT12, a uric acid transporter whose dysfunction elevates blood urate concentration [37], have been identified as key players in uric transport. GLUT12-mediated urate transport is sodium-independent and bidirectional, with enhanced transport activity under acidic pH conditions [37]. Recent structural studies on GLUT9-urate binding have revealed that hydrogen bonds and hydrophobic interactions are crucial for substrate attachment. The carbonyl and amine groups of the imidazole ring, along with the carbonyl group of the pyrimidine ring in uric acid, play vital roles in this process. Our docking and MD simulations indicate similar interaction between GLUT3 and TMZ, involving hydrogen bonds, hydrophobic interactions, and Van der Waals contacts. The carboxamide group on TMZ’s imidazole ring and the amine group of the tetrazine ring are essential for binding. However, GLUT3’s selectivity for glucose and TMZ in GBM cells is not as pronounced as GLUT9 for glucose and uric acid. This may be due to GLUT3’s high affinity and transport capacity for glucose, which is crucial for neurons given the lower glucose levels in their microenvironment [38]. Further research, particularly the crystal structure determination of GLUT3 in different TMZ-binding states, is needed to validate these interactions and elucidate the structural changes during transport. Such insights will enhance our understanding of TMZ cellular uptake and aid in optimizing chemotherapeutic drug design, leveraging GLUT3 transport.

The increased expression of GLUTs allows tumor cells to adapt better to their environment by capturing more glucose, providing them a survival advantage [39]. Numerous studies have investigated this mechanism in tumorigenesis and progression, leading to a growing interest in exploiting this characteristic of tumors through glycoconjugation to target anticancer therapeutics to cancer cells selectively. This approach is inspired by the widespread clinical use of 2-deoxy-2-[^18^F]fluoro-D-glucose (^18^F-FDG), a radiolabeled glucose analog commonly used to visualize tumors and their metastases [40]. The study of glycoconjugates for GLUT1-targeted cancer therapy began in 1995 when Pohl and colleagues introduced glufosfamide [41]. This compound is synthesized by linking β-d-glucose to an alkylated molecule of ifosfamide. Treatment with GLUT1 inhibitors significantly reduced the anticancer efficacy of glufosfamide, supporting its transport through the GLUT1 mechanism [42]. Following the introduction of glufosfamide, various glycoconjugates targeting GLUT1 have been developed. Some of these glycoconjugates were able to suppress the uptake of GLUT1 substrate 2-NBDG (100 μM) by ~20%. MTT assays demonstrated that these compounds were six to eleven times more toxic to cells with high GLUT1 expression than those with low expression. In 2016, He et al reported the conjugation of herbal isolate with glucose through different linkages to identify candidates with low systemic toxicity and high water solubility [43]. Furthermore, using human erythrocytes, Halmos’ group demonstrated that GLUT1 mediates the transport of chlorambucil glycoconjugates, which inhibit [^14^C]glucose uptake in a concentration-dependent manner [44]. However, GLUT1 is widely expressed in various normal tissues, raising concerns about potential side effects of GLUT1-targeted therapies on these tissues. In contrast, GLUT3, while also highly expressed in many tumor cells [42, 45], is predominantly found in neurons and is the primary glucose transporter in the brain. Its more restricted expression pattern in normal tissues suggests that GLUT3-mediated transport of glycoconjugates might be safer than GLUT1-mediated transport, with fewer side effects on normal tissues. Our study demonstrates that GLUT3 can transport TMZ into GBM cells, enhancing chemosensitivity. This highlights a novel role of GLUT3 beyond glucose transport and supports the development of GLUT3-targeted therapies for GBM. Such an in tumor chemosensitivity offers new insights for developing safer and more effective antitumor drugs.

The limited options and significant toxic side effects of chemotherapeutic agents for GBM treatment pose major challenges. CAPE, an oral pro-drug of 5-fluorouracil, is used for various cancers [46–48], showing potential in brain tumor treatment. For instance, the combination of CAPE with other drugs showed intracranial activity and effective disease control in patients with CNS metastasis of breast cancer [49, 50]. Additionally, administering low-dose CAPE 5–7 days before surgery has significantly improved the prognosis of GBM patients [51]. The role of capecitabine transport in its chemosensitivity remains poorly understood. A study assessing the responses of colorectal cancer (CRC) to capecitabine and oxaliplatin regimen found that those with ABCB1 gene variants (rs1128503 and rs1045642) had better disease-free survival rate (DFSR) and overall survival rate (OSR) [52]. This suggests that the efflux of these two chemotherapeutic agents may affect their effectiveness. Although there is a lack of reported studies on capecitabine uptake, research on nucleoside analogs such as gemcitabine, cytarabine (Ara-C), and fludarabine highlights the importance of nucleotide transporters in the efficacy and absorption of these drugs [53, 54]. For example, uptake of gemcitabine is primarily mediated by the human equilibrative nucleoside transporter 1 (hENT1), and cells deficient in hENT1 exhibit high resistance to this nucleoside analogue [53]. The role of hENT1 in gemcitabine sensitivity has been reported in various tumors, including pancreatic cancer [55], meningioma [56], biliary tract cancer (BTC) [57], and non-small cell lung cancer (NSCLC) [58]. In contrast to these studies, our study reveals that GLUT3 can function as a novel nucleoside analog transporter, playing a critical role in sensitizing GBM to CAPE chemotherapy. This lays the theoretical foundation for the clinical use of capecitabine in GBM chemotherapy.

Dietary interventions show great potential for enhancing chemosensitivity and improving cancer treatment outcomes. Strategies like Caloric Restriction (CR), fasting-mimicking diets, Intermittent Fasting (IF), Ketonegenic Diet (KD), and Short-Term Starvation (STS) can influence tumor metabolism and therapy response. CR reduces reactive oxygen species (ROS) to slow tumor growth [59]. Fasting-mimicking diets enhance chemotherapy effectiveness [60–62], while IF increases metabolic flexibility, forcing cancer cells into oxidative phosphorylation and boosting apoptosis [63, 64]. KD induces ketosis, reducing inflammation and glucose supply to cancer cells [65, 66], and STS lowers blood glucose and IGF-1 levels to sensitize tumors to chemotherapy [60]. Our research demonstrates that dietary interventions can further enhance the chemosensitivity of GBM cells overexpressing GLUT3 to TMZ and CAPE. By lowering blood glucose and IGF-1 levels, fasting creates a metabolic environment that increases the uptake of these chemotherapeutic agents by GBM cells. This is particularly significant as it provides a novel approach to improve the efficacy of current GBM treatments. The results of our study not only underscore the importance of GLUT3 in GBM chemotherapy but also offer new insights for designing personalized treatment regimens that integrate dietary interventions with chemotherapy. This approach has the potential to significantly enhance treatment outcomes and improve patient survival rates.

Our study reveals, for the first time, the novel glucose-independent transport function of GLUT3 in the context of GBM chemotherapy. This challenges the common perception that GLUT3 primarily promotes tumor growth through its glucose transport function. Our research also raises several important questions that require further exploration. Understanding the crystal structures of GLUT3 in complexes with TMZ and CAPE in different states will be crucial for gaining insights into the binding and transport mechanisms involved. Are there mechanisms beyond competitive binding that influence GLUT3’s substrate selection? Could other GLUTs also contribute to tumor chemosensitization? Moreover, might other nucleoside analogs be transported specifically by certain GLUTs? Addressing these questions is expected to provide clues for the new use of old antitumor drugs and new strategies for sensitizing tumor chemotherapeutic agents and designing anti-tumor glycoconjugates.

## Materials and methods

### Bioinformatic analysis

Transcriptomic profiles and clinical data of glioma samples, encompassing survival status, age, radiotherapy status, and chemotherapy status, were retrieved from The Cancer Genome Atlas (TCGA) through the UCSC Xena platform. In accordance with the expression levels of *SLC2A1* and *SLC2A3*, GBM patients within the TCGA dataset were stratified into high and low expression groups. To comprehensively explore the potential correlation between GLUT3 (encoded by *SLC2A3*) expression and overall survival (OS) of GBM patients, supplementary data were collected from both TCGA and the Chinese Glioma Genome Atlas (CGGA) databases. Moreover, four glioma cohorts, namely GSE108476, GSE2223, GSE4290, and GSE16011, were obtained from the Gene Expression Omnibus (GEO).

For data preprocessing, the Robust Multichip Average (RMA) algorithm was implemented to perform background correction and normalization on the gene expression data. The original transcript data, which were presented as fragments per kilobase million (FPKM), were subsequently transformed into transcripts per kilobase million (TPM). This conversion was essential for ensuring consistency and facilitating subsequent downstream analyses, enabling more accurate and comparable interpretations of gene expression levels across different datasets.

### Cell culture, plasmid construction, and transfection

All cell lines utilized in this study were procured from the American Type Culture Collection (ATCC). Rigorous testing was conducted, and their identities were authenticated via short tandem repeat (STR) profiling to ensure genetic integrity. The human glioblastoma cell lines U87-MG, U343-MG, U251-MG, and LN-229, along with HEK-293T cells, were cultivated in high-glucose Dulbecco’s Modified Eagle Medium (DMEM; containing 25 mM glucose, 4 mM glutamine, and 110 mg/L pyruvate) supplemented with 10% fetal bovine serum (FBS, TransSerum). The cells were maintained at 37 °C within a humidified incubator with a 5% CO_2_ atmosphere.

For the construction of GLUT3 knockdown plasmids, the designed GLUT3-specific short hairpin RNA (shRNA) sequences were cloned into the pLKO.1-puro vector, which had been linearized using AgeI and EcoRI restriction enzymes. A non-targeting sequence was employed as a negative control. For GLUT3 overexpression plasmids, the coding DNA sequence (CDS) of GLUT3 was amplified through polymerase chain reaction (PCR) and subsequently cloned into the pCDH-puro vector. The GLUT3 mutant expression plasmids were generated via site-directed mutagenesis. Specifically, the Q5 Site-Directed Mutagenesis Kit (E0554S, New England BioLabs) was utilized according to the manufacturer’s protocol, with the pCDH-puro-GLUT3 plasmid serving as the template. For the GLUT3-GFP plasmid used in the microscale thermophoresis (MST) assay, the green fluorescent protein (GFP) coding sequence was fused to the C-terminal of GLUT3. An appropriate linker sequence was inserted between GLUT3 and GFP to ensure an optimal spatial configuration, preventing potential steric hindrance. All plasmids employed in the experiments were subjected to DNA sequencing to confirm their correct construction. The specific sequences of the shRNAs were as follows:

- sh-Scramble (NC):5′-TTCTCCGAACGTGTCACGT-3′
- sh-GLUT3-1:5′-GCTTGGTCTTTGTAGCCTTCTT-3′
- sh-GLUT3-2:5′-AGTAGCTAAGTCGGTTGAAAT-3′

The recombinant constructs, as well as assistant vectors psPAX2 and pMD2.G, were co-transfected into HEK-293T cells. Viral supernatants were collected 48 h later, clarified by filtration through 0.45 μm filters. Virus-containing supernatants were mixed with PEG 8000 and left at rest overnight. This was followed by centrifugation at 4000 rpm at 4 °C for 45 min. The supernatant was discarded, and the precipitate was resuspended in fresh medium before mixing with 10 μg/ml polybrene (Millipore) and incubated with target cells for another 24 h. Transduced cells were selected using puromycin (2 mg/ml) for at least 5 days. Knockdown and overexpression efficiencies were quantified by qPCR.

The recombinant plasmids, along with the helper vectors psPAX2 and pMD2. G was co-transfected into HEK-293T cells. 48 h post-transfection, the viral supernatants were collected and filtered through 0.45 μm membrane filters to remove cellular debris. The virus-containing filtrates were then mixed with polyethylene glycol 8000 (PEG 8000) and allowed to stand overnight at 4 °C to promote virus precipitation. Subsequently, the mixtures were centrifuged at 4000 rpm for 45 min at 4 °C. The supernatants were discarded, and the resulting viral pellets were resuspended in fresh culture medium. The resuspended virus was then combined with 10 μg/ml polybrene (Millipore) and incubated with the target cells for an additional 24 h. Transduced cells were selected using puromycin (2 mg/ml) for a period of at least 5 days. The knockdown and overexpression efficiencies of GLUT3 were quantified by quantitative real-time polymerase chain reaction (qPCR).

### RNA extraction and quantitative real-time PCR analysis

Total RNA was extracted from different groups of GBM cells using the TRIzol reagent (Invitrogen) according to the manufacturer’s instructions. The purity and concentration of the extracted RNA were assessed using a NanoDrop spectrophotometer (Thermo Fisher Scientific). Subsequently, the RNA samples were reverse-transcribed into cDNA using the ReverTra Ace® qPCR RT Master Mix with gDNA Remover Kit (TOYOBO, Japan). Quantitative real-time PCR (qPCR) was performed using SYBR Green PCR Mix (Q111-02, Vazyme) on an ABI PRISM 7500 Real-Time PCR System (Applied Biosystems, Foster City, CA, USA). The thermal cycling conditions were as follows: initial denaturation at 95 °C for 30 s, followed by 40 cycles of 95 °C for 5 s and 60 °C for 30 s. The relative expression levels of the genes of interest were calculated using the comparative Ct method (2^−ΔΔCt^), with Actin serving as the internal control. The sequences of the primers used in this study are listed in Table S1.

### Protein extraction and western blot analysis

Protein samples were prepared by lysing the cells and quantifying the protein concentration using the Bradford assay. The lysates were then mixed with SDS loading dye and subjected to SDS-PAGE. After electrophoresis, the proteins were transferred onto polyvinylidene difluoride (PVDF) membranes. The membranes were blocked with 5% non-fat dry milk in 1X Tris-buffered saline containing 0.1% Tween 20 (TBS-T) for 1 h at room temperature (RT). Subsequently, the membranes were incubated overnight at 4 °C with the following primary antibodies: anti-GLUT3 (Abcam, ab191071) and β-actin (Proteintech, 66009-1-Ig). After washing with 1X TBS-T, the membranes were incubated with secondary antibodies conjugated with horseradish peroxidase (anti-rabbit or anti-mouse) for 1 h at RT. The membranes were then washed thoroughly, and the protein bands were visualized using a Gel Imaging System (BINTA2020D) according to the manufacturer’s instructions.

### Cell viability assay

Cell viability was assessed using the Cell Counting Kit-8 (CCK-8) assay. Glioblastoma (GBM) cells were seeded into 96-well plates at a density of ~3 × 10^3^ cells per well, with three replicate wells per group. The total volume in each well was adjusted to 100 µL. After allowing the cells to adhere and grow, 10 µL of CCK-8 reagent was added to each well 1 h prior to the target detection time. The plates were then incubated for an additional 1 h at 37 °C. The absorbance (optical density, OD) was measured at 450 nm using a microplate reader. Data were collected at 6, 24, 48, 72, and 96 h post-seeding, with measurements taken at the same time each day.

### Migration and invasion assays

Transwell inserts with an 8 µm pore size (0216, BD Biosciences) were placed into a 24-well culture plate. For the invasion assay, Transwell inserts were coated with Matrigel (354234, Corning), while uncoated inserts were used for the migration assay. GBM cells were resuspended in DMEM without fetal bovine serum (FBS) at a density of 3 × 10^4^ cells per well and seeded into the upper chambers. The lower chambers were filled with 600 µL of DMEM containing 10% FBS as a chemoattractant. After incubation for 24–48 h, cells that had migrated or invaded to the lower surface of the Transwell inserts were fixed and stained with 0.1% crystal violet. The stained cells were then photographed using a light microscope. Each experimental condition was performed in triplicate wells.

### Wound healing assay

GBM cells were seeded into each well of a 6-well plate at a density of 2 × 10^6^ cells per well and incubated overnight in a humidified incubator at 37 °C with 5% CO_2_. A sterile 10 µL pipette tip was used to create a consistent wound in the form of a “#“ shape across the monolayer of cells. Following scratching, PBS was added to the wells, and the plate was gently agitated to dislodge any detached cells. The PBS was then removed, and this step was repeated three times to ensure the removal of cells dislodged by the scratch. Subsequently, fresh serum-free medium was added to the wells, and the plate was returned to the incubator at 37 °C with 5% CO_2_.

Images of three randomly selected fields per group were captured at 0, 24, and 48 h using an inverted microscope at the same position for each time point. The area of the scratches was measured at each time point using ImageJ software to quantify the wound closure.

### Colony formation assay and drug treatment

GBM cells were harvested using 0.25% trypsin when cell viability was confirmed to be optimal, and the cell concentration was determined using a hemocytometer. A total of 1 × 10³ cells were seeded into each well of a 6-well plate and incubated at 37 °C overnight. The following day, cell morphology was examined under an inverted microscope. The culture medium was replaced every 2 days, and cell growth was monitored regularly. When cell colonies exceeded 50 cells, they were recorded. The cells were then washed with pre-warmed PBS. Subsequently, the cells were fixed with 4% paraformaldehyde (1 mL per well) for 20 min at room temperature. After fixation, the cells were washed three times with PBS. A total of 1 mL of 0.1% crystal violet staining solution was added to each well for 20 min. The wells were then washed three times with distilled water, covered with a plate lid, and left to dry overnight. The number of colonies in each well was quantified using ImageJ software.

To investigate the effects of drugs on cell proliferation, GBM cells were treated with fresh medium containing temozolomide (200 µM) or capecitabine (400 µM). The medium was replaced every 2 days. When colonies visible to the naked eye had formed, the cells were fixed with 4% paraformaldehyde and stained with 0.1% crystal violet as described above. The colonies were then quantified using ImageJ software for statistical analysis.

### IC50 determination

The GBM cell line was seeded into a 96-well plate at a density of 5 × 10^3^ cells per well and incubated at 37 °C in a humidified atmosphere containing 5% CO_2_ overnight. After 24 h of incubation, the cells were treated with varying concentrations of temozolomide or capecitabine. Following 24 h of drug treatment, 10 µL of CCK-8 reagent was added to each well, and the cells were incubated for an additional 1 h at 37 °C. The absorbance was then measured at 450 nm using a microplate reader.

In this study, the experimental groups were designated as Group A (cells treated with temozolomide (TMZ) or capecitabine (CAPE)), the control group as Group B (cells treated with DMSO), and the blank control group as Group C (untreated cells). The cell viability rate was calculated using the formula: (A-C)/(B-C) × 100%, where A, B, and C represent the absorbance values of Group A, Group B, and Group C, respectively.

### Determination of CC50 values

The human normal astrocyte cell line SVGp12 was seeded in a 96-well plate at a density of 5 × 10^3^ cells per well and incubated overnight at 37 °C in a humidified atmosphere containing 5% CO_2_. Subsequently, the cells were then treated with varying concentrations of TMZ or CAPE for 24 h. After the treatment period, 10 μL of CCK-8 reagent was added to each well, followed by an additional incubation for 1 h under the same conditions. The absorbance at 450 nm was measured using a microplate reader. In this study, the experimental group treated with TMZ or CAPE was designated as group A, the control group treated with DMSO as group B, and the blank control group without cells as group C. The cell viability rate was calculated using the formula: (A-C)/(B-C) × 100%, where A, B, and C represent the absorbance values of group A, group B, and group C, respectively. Each experiment was performed in triplicate, and the CC50 values were determined as the drug concentration required to reduce cell viability by 50% relative to the control group.

### 2-NBDG uptake assay

Cellular glucose uptake was assessed using the fluorescent glucose analog 2-NBDG. GBM cells were seeded into 96-well plates at a density of 4 × 10^4^ cells per well and allowed to adhere for 24 h. After 6–12 h, the culture medium (containing 10% fetal bovine serum, FBS) was removed, and the cells were incubated in 100 µL of glucose-free DMEM medium for 1 h at 37 °C in a humidified atmosphere containing 5% CO_2_. The glucose-free medium was then replaced with 100 µL of DMEM containing 100 µM 2-NBDG, and the cells were incubated for an additional 30 min at 37 °C. Following incubation, the cells were washed three times with cold PBS to remove unincorporated 2-NBDG. The fluorescence intensity was immediately measured using a Synergy™ H1 automatic microplate reader with excitation and emission wavelengths set at 485 nm and 528 nm, respectively.

To investigate the effects of TMZ and CAPE on glucose uptake via GLUT3, a concentration-dependent competition assay was performed. GBM cells were treated with varying concentrations of TMZ (1, 10, and 100 µM) or CAPE (1, 10, and 100 µM) in glucose-free DMEM medium containing 100 µM 2-NBDG. After 30 min of incubation at 37 °C, the cells were washed three times with cold PBS, and the fluorescence intensity was measured as described above. The overall glucose uptake was quantified by measuring the fluorescence intensity, and the results were normalized using Hoechst staining to account for differences in cell number.

### Molecular docking and structural analysis

The two-dimensional structure of the compound was constructed by ChemDraw and then imported into ChemDraw 3D. The MM2 module was employed to minimize the molecular energy, and the conformation with the lowest energy was obtained and saved as a mol2 file.

The GLUT3 protein structure (PDB ID 4ZW9) was retrieved from the Protein Data Bank (PDB; https://www.rcsb.org) database and visualized using PyMOL. For docking preparation, both the ligand (compound) and receptor were saved as pdbqt by dehydration, hydrogenation, calculation of charge, and incorporation of non-polar hydrogen using Mgtools 1.5.6.

Molecular docking was performed using AutoDock Vina 1.1.2. The binding interactions between the ligand and the receptor were evaluated, and the docking poses with the highest scores were selected for further analysis. The selected poses were visualized using PyMOL and Discovery Studio to assess the binding modes and interactions in detail.

### Molecular dynamics (MD) simulation

This study employed the GROMACS 5.1.5 software package to perform MD simulations. The GROMOS54a7 force field was utilized for the protein and lipid parameters, while the SPCE model was used for water molecules. Long-range electrostatic interactions were handled using the Particle Mesh Ewald (PME) method. The system temperature and pressure were maintained using the V-rescale thermostat and the Parrinello-Rahman barostat, respectively.

The protein complex was embedded in a 1,2-dipalmitoyl-sn-glycero-3-phosphocholine (DPPC) lipid bilayer containing 128 lipid molecules. Neutralizing ions were added to ensure the system was electrically neutral, with a salt concentration of 0.145 M. The phospholipid bilayer was expanded by a factor of 4 using the inflateGro.PL script, followed by energy minimization to relieve any steric clashes or unfavorable interactions. The initial structure of the transmembrane protein was obtained, and counterions were added to neutralize the system.

Energy minimization was performed using the steepest descent algorithm. This was followed by a 100 ps restrained isothermal equilibration simulation to bring the system to a temperature of 300 K and a pressure of 1 atmosphere. Subsequently, a 1 ns restrained isobaric equilibration simulation was conducted. The coupling constants for temperature and pressure were set to 0.2 ps. Finally, a 100 ns free MD simulation was performed to allow the system to evolve spontaneously.

The integration time step used in the simulation was 2 fs, and the trajectory was saved every 2 ps for subsequent data analysis. The equilibrium state of the system was assessed by analyzing the root mean squared deviation (RMSD) and radius of gyration (Rg) of the protein complex over time. The binding free energy was estimated using the g_mmpbsa module, which calculates the MM-PBSA (Molecular Mechanics-Poisson Boltzmann Surface Area) binding free energy.

### Microscale thermophoresis (MST) assay

The binding affinities of GLUT3 and its mutants with various ligands (glucose, TMZ, and CAPE) were assessed using MST. Cell lysates from U87 and U343 cells stably expressing GFP-tagged wild-type GLUT3 or its mutants were utilized as the fluorescence source, while lysates from cells expressing GFP alone served as controls. The cell lysates were diluted at least 10-fold in the assay buffer (10 mM sodium phosphate buffer, pH 7.4, 1 mM MgCl_2_, 3 mM KCl, 150 mM NaCl, 1% Tween-20) to achieve optimal fluorescence intensity.

For each measurement, the diluted cell lysates were mixed with an equal volume of ligands at 16 different serially diluted concentrations and incubated at room temperature for 5 min. Both the cell lysates and ligands were diluted and dissolved in the same pH-gradient assay buffer (pH 6.8 and 7.4). MST measurements were performed in standard treated capillaries (K002) at 37 °C, with 40% LED power and high MST power settings.

Data from three independent measurements were analyzed using MO. Affinity Analysis software (version 2.1.3, NanoTemper Technologies). The binding affinity was evaluated using the signal from an MST-on time of 2.5 s, and the dissociation constant (*K*_d_) was determined by fitting the data to a binding model.

### Cellular uptake assay of TMZ and CAPE using LC-MS/MS

The cellular uptake of TMZ and CAPE was measured using LC-MS/MS with the internal standards ^2^H_3_-TMZ and ^2^H_11_-CAPE, respectively. A total of 6 × 10^6^ cells were seeded into each well of a 6 cm Petri dish and cultured at 37 °C in a humidified atmosphere containing 5% CO_2_ for 8 h. The cells were then washed twice with PBS (137 mM NaCl, 2.7 mM KCl, 10 mM Na_2_HPO_4_, 1.8 mM KH_2_PO_4_, pH 7.4) and incubated in transport buffer (140 mM NaCl, 5 mM KCl, 2 mM MgCl_2_, 2 mM CaCl_2_, 30 mM Tris-HCl, pH 7.4) for 15 min at 37 °C. Subsequently, the cells were incubated in 3 mL of transport buffer (pH 4.5 and pH 7.4) containing 6 µL of TMZ (pH 4.5) and CAPE (pH 7.4) (final concentration 200 µM) for 15 min at 37 °C. For the concentration-dependent uptake assay, excess label-free glucose (1, 10, and 25 mM) was used to compete for GLUT3 binding. Cell supernatants were collected and stored at 4 °C for subsequent extraction. The harvested cells were washed three times with PBS and extracted with 500 µL of 80% methanol for 30 min at −80 °C. The cell lysate was then centrifuged at 14,000 × *g* for 5 min at 4 °C, followed by 2 min of sonication. The supernatant was transferred to a new centrifuge tube. For LC-MS/MS analysis, the cell supernatants and cell lysates were diluted at a specific ratio. A total of 100 µL of each sample was mixed with 100 µL of acetonitrile (or standard reagent) and 300 µL of internal standard solution (^2^H_3_-TMZ 100 ng/mL and ^2^H_11_-CAPE 20 ng/mL dissolved in acetonitrile). Standard mixtures of TMZ and CAPE at various concentrations (0, 0.1, 0.15, 0.2, 0.25, 0.3, 0.5, 0.75, 0.9, 1, 2, 3, 3.2, and 4 µM) were prepared in acetonitrile. Quality control (QC) samples were prepared and analyzed alongside the experimental samples to monitor the stability and repeatability of the instrument. QC samples were inserted regularly and analyzed every 6–12 samples. Each experiment was repeated 5–6 times.

### LC-MS/MS analysis

LC-MS/MS analyses were performed using a high-performance liquid chromatography (HPLC) system (Shimadzu, LC-20A) coupled to a quadrupole time-of-flight mass spectrometer (ABSciex, API4000 QTRAP). The samples were separated on a 2.1 × 100 mm ACQUITY Premier BEH C18 1.7 µm VanGuard™ FIT column (Waters, Ireland). The mobile phases consisted of: Mobile phase A (0.1% formic acid and 2.5 mM ammonium acetate in water) and Mobile phase B (0.1% formic acid and 2.5 mM ammonium acetate in acetonitrile). Gradient elution was employed, with the flow rates and gradient conditions detailed in Table S2.

The mass spectrometry conditions were as follows: Ion source: electrospray ionization (ESI); Detection mode: positive ion mode; Scanning method: multiple reaction monitoring (MRM); Spray voltage (IS): 5500 V; Ion source temperature (TEM): 555 °C; Gas curtain pressure (CUR): 25 psi; Collision activation dissociation (CAD): MEDIUM; Nebulizer gas pressure (GS1): 55 psi; Auxiliary gas pressure (GS2): 55 psi. The qualitative and quantitative ion pairs, along with the detailed mass spectrometric parameters, are listed in Table S3.

The quantitative analysis of the LC-MS/MS data was performed using the Analyst 1.6 software.

### Quantification of TMZ and CAPE levels in nude mouse tumors by LC-MS/MS

TMZ and CAPE levels in tumors from nude mice were measured using LC-MS/MS. Tumor tissues were excised from the mice and homogenized. The homogenized samples were then centrifuged at maximum speed (~14,000 × *g*) at 4 °C for 10 min. The supernatant was collected and transferred to a new centrifuge tube.

For LC-MS/MS analysis, the supernatants were diluted to an appropriate concentration. A total of 100 µL of each sample was mixed with 100 µL of acetonitrile (or standard reagent) and 300 µL of internal standard solution (^2^H_3_-TMZ 100 ng/mL and ^2^H_11_-CAPE 20 ng/mL dissolved in acetonitrile). Standard mixtures of TMZ and CAPE at various concentrations were prepared in acetonitrile. To ensure the stability and repeatability of the instrument analysis, QC samples were prepared and analyzed alongside the experimental samples. QC samples were inserted regularly and analyzed every 6–12 samples. Each experiment was repeated 6 times.

LC-MS/MS analyses were performed using an HPLC system (Shimadzu, LC-20A) coupled to a SCIEX Triple Quad™ 7500 mass spectrometer. The samples were separated on a Syncronis HILIC column (2.1 × 100 mm, 1.7 µm; Thermo Scientific). The mobile phases consisted of: Mobile phase A (0.1% formic acid and 2.5 mM ammonium acetate in water) and Mobile phase B (0.1% formic acid and 2.5 mM ammonium acetate in acetonitrile). Gradient elution was employed, with the flow rates and gradient conditions detailed in Table S4.

The mass spectrometry conditions were as follows: Ion source: electrospray ionization (ESI); Detection mode: positive ion mode; Scanning method: multiple reaction monitoring (MRM); Spray voltage (IS): 5500 V; Ion source temperature (TEM): 555 °C; Gas curtain pressure (CUR): 25 psi; Collision activation dissociation (CAD): MEDIUM; Nebulizer gas pressure (GS1): 55 psi; Auxiliary gas pressure (GS2): 55 psi. The qualitative and quantitative ion pairs, along with the detailed mass spectrometric parameters, are listed in Table S5.

The SCIEX OS software was used to process the LC-MS/MS data for quantitative analysis.

### Tumor xenografts

All animal studies were approved and supervised by the Ethics Committee for Animal Use and Care at Peking University. Nude mice (BALB/c-nu) were purchased and maintained as previously described. For the establishment of xenograft tumor models, 1 × 10^6^ U251-MG cells (either negative vector control or overexpressing GLUT3) were injected into the bilateral axillary fossae of 4-week-old nude mice. After 7 days, when palpable tumors had formed, the mice were randomly divided into six experimental groups (*n* = 7 per group), and treatment was initiated immediately. The treatment groups were as follows:

1. Control: Saline administered intraperitoneally (i.p.) every 3 days.
2. Control-fasting: Mice were fasted for 1 day before receiving saline (i.p.) every 3 days.
3. TMZ: TMZ (40 mg/kg) administered intraperitoneally (i.p.) every 3 days.
4. TMZ-fasting: Mice were fasted for 1 day before receiving TMZ (40 mg/kg, i.p.) every 3 days.
5. CAPE: CAPE (20 mg/kg) administered intraperitoneally (i.p.) every 3 days.
6. CAPE-fasting: Mice were fasted for 1 day before receiving CAPE (20 mg/kg, i.p.) every 3 days.

Tumor diameters were measured every 4 days using calipers. At the end of the study, all mice were sacrificed, and the tumors were carefully excised and weighed. Tumor volume was calculated using the formula *V* = (length*width^2^)/2. Data were analyzed using statistical software to evaluate the differences among groups.

### Blood glucose detection in nude mice

All animal experiments were conducted with the approval and supervision of the Ethics Committee for Animal Use and Care at Peking University. Male and female BALB/c-nu nude mice were purchased and acclimatized for one week before the experiment, as previously described.

For the blood glucose detection experiments, 24 male and female BALB/c-nu nude mice were each randomly divided into two groups: a control group maintained on a standard diet and a fasting group subjected to 24 h of fasting. Blood glucose levels were measured using a 27 G needle to puncture the lateral tail vein. The first drop of blood was discarded to avoid contamination, and gentle pressure was applied to the base of the tail to generate a 2–3 µL blood droplet. The test strip was vertically contacted with the blood droplet, and the blood glucose value (mmol/L) was recorded within 45 s. Each measurement was repeated three times to ensure accuracy. Data were analyzed using statistical software to assess significant differences between the groups.

### Immunohistochemistry (IHC) staining and analysis

Primary antibodies were applied at a dilution of 1:200. For immunostaining, secondary antibodies conjugated with peroxidase were used, specifically goat anti-mouse or goat anti-rabbit, following the manufacturer’s instructions. The IHC images were analyzed to calculate the mean value of the integral optical density (IOD) using ImagePro Plus 6.0 software.

### Glioma tissue microarray immunohistochemistry and analysis

Tissue microarrays (TMAs) comprising 80 cases of human glioma and adjacent normal brain tissues were obtained from Shanghai Superbiotek Pharmaceutical Technology Company. For antigen retrieval, slides were treated with citrate buffer (pH 6.0) and heated in a microwave for 15 min. Endogenous peroxidase activity was blocked using 3% hydrogen peroxide. Sections were incubated overnight at 4 °C with anti-GLUT3 antibody (Abcam, ab41525) at a 1:200 dilution. After washing, slides were incubated with a biotinylated secondary antibody for 30 min at room temperature. Staining was visualized with diaminobenzidine (DAB), and sections were counterstained with hematoxylin. Slides were dehydrated, cleared, and mounted with coverslips.

Quantitative analysis was conducted using ImagePro Plus 6.0 software. The integrated optical density (IOD) of GLUT3 staining was measured, and GLUT3 expression levels were determined for each image based on IOD values. Damaged samples were excluded from analysis to ensure accuracy. This method enabled an objective, quantitative evaluation of GLUT3 protein expression in viable tissue samples.

### Statistical analysis

Data from at least three independent biological replicates were statistically analyzed using GraphPad Prism and IBM SPSS to assess intergroup differences. Two-tailed Student’s *t*-test was used to assess statistical significance for comparisons between two groups, while one-way ANOVA followed by Tukey’s post-hoc test was applied for comparisons involving three groups. A *P*-value of less than 0.05 was considered statistically significant. The following notation was used: ns (not significant), *P < 0.05, **P < 0.01, ***P < 0.001.

## Supplementary information

Table S1

Table S2

Table S3

Table S4

Table S5

Supplementary Figures and Legends

Original Western blot

## Data Availability

All data and materials are available in the main text.

## References

[1] Heuer S, Winkler F. Glioblastoma revisited: from neuronal-like invasion to pacemaking. Trends Cancer. 2023;9:887–96. 37586918 10.1016/j.trecan.2023.07.009

[2] Zhao M, van Straten D, Broekman MLD, Préat V, Schiffelers RM. Nanocarrier-based drug combination therapy for glioblastoma. Theranostics. 2020;10:1355–72. 31938069 10.7150/thno.38147 PMC6956816

[3] Shergalis A, Bankhead A 3rd, Luesakul U, Muangsin N, Neamati N. Current challenges and opportunities in treating glioblastoma. Pharm Rev. 2018;70:412–45. 29669750 10.1124/pr.117.014944 PMC5907910

[4] Stupp R, Mason WP, van den Bent MJ, Weller M, Fisher B, Taphoorn MJ, et al. Radiotherapy plus concomitant and adjuvant temozolomide for glioblastoma. N Engl J Med. 2005;352:987–96. 15758009 10.1056/NEJMoa043330

[5] Agarwala SS, Kirkwood JM. Temozolomide, a novel alkylating agent with activity in the central nervous system, may improve the treatment of advanced metastatic melanoma. Oncologist. 2000;5:144–51. 10794805 10.1634/theoncologist.5-2-144

[6] Sarkaria JN, Kitange GJ, James CD, Plummer R, Calvert H, Weller M, et al. Mechanisms of chemoresistance to alkylating agents in malignant glioma. Clin Cancer Res. 2008;14:2900–8. 18483356 10.1158/1078-0432.CCR-07-1719 PMC2430468

[7] Lee SY. Temozolomide resistance in glioblastoma multiforme. Genes Dis. 2016;3:198–210. 30258889 10.1016/j.gendis.2016.04.007 PMC6150109

[8] Rao V, Kumar G, Vibhavari RJA, Nandakumar K, Thorat ND, Chamallamudi MR, et al. Temozolomide resistance: a multifarious review on mechanisms beyond O-6-methylguanine-dna methyltransferase. CNS Neurol Disord Drug Targets. 2023;22:817–31. 35379142 10.2174/1871527321666220404180944

[9] Singh N, Miner A, Hennis L, Mittal S. Mechanisms of temozolomide resistance in glioblastoma - a comprehensive review. Cancer Drug Resist. 2021;4:17–43. 34337348 10.20517/cdr.2020.79 PMC8319838

[10] Ortiz R, Perazzoli G, Cabeza L, Jiménez-Luna C, Luque R, Prados J, et al. Temozolomide: an updated overview of resistance mechanisms, nanotechnology advances and clinical applications. Curr Neuropharmacol. 2021;19:513–37. 32589560 10.2174/1570159X18666200626204005 PMC8206461

[11] Xie Y, He L, Zhang Y, Huang H, Yang F, Chao M, et al. Wnt signaling regulates MFSD2A-dependent drug delivery through endothelial transcytosis in glioma. Neuro Oncol. 2023;25:1073–84. 36591963 10.1093/neuonc/noac288 PMC10237416

[12] Ramalho MJ, Andrade S, Coelho MAN, Loureiro JA, Pereira MC. Biophysical interaction of temozolomide and its active metabolite with biomembrane models: the relevance of drug-membrane interaction for Glioblastoma Multiforme therapy. Eur J Pharm Biopharm. 2019;136:156–63. 30682492 10.1016/j.ejpb.2019.01.015

[13] Rubio-Camacho M, Encinar JA, Martínez-Tomé MJ, Esquembre R, Mateo CR. The interaction of temozolomide with blood components suggests the potential use of human serum albumin as a biomimetic carrier for the drug. Biomolecules. 2020;10:1015. 10.3390/biom10071015 PMC7408562 32659914

[14] Zhuang Y, Zhao J, Xu X, Bi L. Downregulation of GLUT3 promotes apoptosis and chemosensitivity of acute myeloid leukemia cells via EGFR signaling. Arch Iran Med. 2018;21:73–8. 29664658

[15] Li X, Zhao G, Mi X, Xu T, Li X, Liu B. Ajuba overexpression promotes breast cancer chemoresistance and glucose uptake through TAZ-GLUT3/survivin pathway. Biomed Res Int. 2022;2022:3321409. 35178446 10.1155/2022/3321409 PMC8844350

[16] Ali R, Alhaj Sulaiman A, Memon B, Pradhan S, Algethami M, Aouida M, et al. Altered regulation of the glucose transporter GLUT3 in PRDX1 null cells caused hypersensitivity to arsenite. Cells. 2023;12:2682. 10.3390/cells12232682 PMC10705171 38067110

[17] Chen K, Li T, Diao H, Wang Q, Zhou X, Huang Z, et al. SIRT7 knockdown promotes gemcitabine sensitivity of pancreatic cancer cells via upregulation of GLUT3 expression. Cancer Lett. 2024;598:217109. 39002692 10.1016/j.canlet.2024.217109

[18] Luo W, Schork NJ, Marschke KB, Ng SC, Hermann TW, Zhang J, et al. Identification of polymorphisms associated with hypertriglyceridemia and prolonged survival induced by bexarotene in treating non-small cell lung cancer. Anticancer Res. 2011;31:2303–11. 21737656

[19] Tooker P, Yen WC, Ng SC, Negro-Vilar A, Hermann TW. Bexarotene (LGD1069, Targretin), a selective retinoid X receptor agonist, prevents and reverses gemcitabine resistance in NSCLC cells by modulating gene amplification. Cancer Res. 2007;67:4425–33. 17483357 10.1158/0008-5472.CAN-06-4495

[20] Holman GD. Structure, function and regulation of mammalian glucose transporters of the SLC2 family. Pflug Arch. 2020;472:1155–75. 10.1007/s00424-020-02411-3 PMC7462842 32591905

[21] Wuest M, Trayner BJ, Grant TN, Jans HS, Mercer JR, Murray D, et al. Radiopharmacological evaluation of 6-deoxy-6-[18 F]fluoro-D-fructose as a radiotracer for PET imaging of GLUT5 in breast cancer. Nucl Med Biol. 2011;38:461–75. 21531283 10.1016/j.nucmedbio.2010.11.004

[22] Zhang S, Wang X, Zhang R, Cui Y, Zhang H, Song W, et al. A GLUT1 inhibitor-based fluorogenic probe for Warburg effect-targeted drug screening and diagnostic imaging of hyperglycolytic cancers. Anal Chim Acta. 2021;1167:338593. 34049629 10.1016/j.aca.2021.338593

[23] Boado RJ, Black KL, Pardridge WM. Gene expression of GLUT3 and GLUT1 glucose transporters in human brain tumors. Brain Res Mol Brain Res. 1994;27:51–7. 7877454 10.1016/0169-328x(94)90183-x

[24] Handa RK, DeJoseph MR, Singh LD, Hawkins RA, Singh SP. Glucose transporters and glucose utilization in rat brain after acute ethanol administration. Metab Brain Dis. 2000;15:211–22. 11206590 10.1007/BF02674530

[25] Arora A, Somasundaram K. Glioblastoma vs temozolomide: can the red queen race be won?. Cancer Biol Ther. 2019;20:1083–90. 31068075 10.1080/15384047.2019.1599662 PMC6606031

[26] Lundy DJ, Nguyễn H, Hsieh PCH. Emerging nano-carrier strategies for brain tumor drug delivery and considerations for clinical translation. Pharmaceutics. 2021;13:1193. 10.3390/pharmaceutics13081193 PMC8399364 34452156

[27] Mohammad SN, Hopfinger AJ. Chemical reactivity of a methyldiazonium ion with nucleophilic centers of DNA bases. J Theor Biol. 1980;87:401–9. 7230851 10.1016/0022-5193(80)90367-7

[28] Ghosh D, Nandi S, Bhattacharjee S. Combination therapy to checkmate glioblastoma: clinical challenges and advances. Clin Transl Med. 2018;7:33. 30327965 10.1186/s40169-018-0211-8 PMC6191404

[29] Chinnasamy D, Fairbairn LJ, Neuenfeldt J, Treisman JS, Hanson JP Jr, Margison GP, et al. Lentivirus-mediated expression of mutant MGMTP140K protects human CD34+ cells against the combined toxicity of O6-benzylguanine and 1,3-bis(2-chloroethyl)-nitrosourea or temozolomide. Hum Gene Ther. 2004;15:758–69. 15319033 10.1089/1043034041648417

[30] Vlachostergios PJ, Hatzidaki E, Papandreou CN. MGMT repletion after treatment of glioblastoma cells with temozolomide and O6-benzylguanine implicates NFkappaB and mutant p53. Neurol Res. 2013;35:879–82. 23561593 10.1179/1743132813Y.0000000191

[31] Wu M, Song D, Li H, Ahmad N, Xu H, Yang X, et al. Resveratrol enhances temozolomide efficacy in glioblastoma cells through downregulated MGMT and negative regulators-related STAT3 inactivation. Int J Mol Sci. 2023;24:9453. 10.3390/ijms24119453 PMC10253519 37298405

[32] Haemmig S, Baumgartner U, Glück A, Zbinden S, Tschan MP, Kappeler A, et al. miR-125b controls apoptosis and temozolomide resistance by targeting TNFAIP3 and NKIRAS2 in glioblastomas. Cell Death Dis. 2014;5:e1279. 24901050 10.1038/cddis.2014.245 PMC4611719

[33] Yang T, Zhang N, Liu Y, Yang R, Wei Z, Liu F, et al. Nanoplatelets modified with RVG for targeted delivery of miR-375 and temozolomide to enhance gliomas therapy. J Nanobiotechnol. 2024;22:623. 10.1186/s12951-024-02895-6 PMC11476726 39402578

[34] Xiong J, Guo G, Guo L, Wang Z, Chen Z, Nan Y, et al. Amlexanox enhances temozolomide-induced antitumor effects in human glioblastoma cells by inhibiting IKBKE and the Akt-mTOR signaling pathway. ACS Omega. 2021;6:4289–99. 33644550 10.1021/acsomega.0c05399 PMC7906592

[35] Yan Y, Liu Y, Liang Q, Xu Z. Drug metabolism-related gene ABCA1 augments temozolomide chemoresistance and immune infiltration abundance of M2 macrophages in glioma. Eur J Med Res. 2023;28:373. 37749600 10.1186/s40001-023-01370-6 PMC10518970

[36] Lustig SD, Kodali SK, Longo SL, Kundu S, Viapiano MS. Ko143 reverses MDR in glioblastoma via deactivating p-glycoprotein, sensitizing a resistant phenotype to TMZ treatment. Anticancer Res. 2022;42:723–30. 35093870 10.21873/anticanres.15530

[37] Toyoda Y, Takada T, Miyata H, Matsuo H, Kassai H, Nakao K, et al. Identification of GLUT12/SLC2A12 as a urate transporter that regulates the blood urate level in hyperuricemia model mice. Proc Natl Acad Sci USA. 2020;117:18175–7. 32690690 10.1073/pnas.2006958117 PMC7414087

[38] Simpson IA, Dwyer D, Malide D, Moley KH, Travis A, Vannucci SJ. The facilitative glucose transporter GLUT3: 20 years of distinction. Am J Physiol Endocrinol Metab. 2008;295:E242–53. 18577699 10.1152/ajpendo.90388.2008 PMC2519757

[39] Szablewski L. Expression of glucose transporters in cancers. Biochim Biophys Acta. 2013;1835:164–9. 23266512 10.1016/j.bbcan.2012.12.004

[40] Som P, Atkins HL, Bandoypadhyay D, Fowler JS, MacGregor RR, Matsui K, et al. A fluorinated glucose analog, 2-fluoro-2-deoxy-D-glucose (F-18): nontoxic tracer for rapid tumor detection. J Nucl Med. 1980;21:670–5. 7391842

[41] Pohl J, Bertram B, Hilgard P, Nowrousian MR, Stüben J, Wiessler M. D-19575-a sugar-linked isophosphoramide mustard derivative exploiting transmembrane glucose transport. Cancer Chemother Pharmacol. 1995;35:364–70. 7850916 10.1007/s002800050248

[42] Calvaresi EC, Hergenrother PJ. Glucose conjugation for the specific targeting and treatment of cancer. Chem Sci. 2013;4:2319–33. 24077675 10.1039/C3SC22205E PMC3784344

[43] He QL, Minn I, Wang Q, Xu P, Head SA, Datan E, et al. Targeted delivery and sustained antitumor activity of triptolide through glucose conjugation. Angew Chem Int Ed Engl. 2016;55:12035–9. 27574181 10.1002/anie.201606121

[44] Halmos T, Santarromana M, Antonakis K, Scherman D. Synthesis of glucose-chlorambucil derivatives and their recognition by the human GLUT1 glucose transporter. Eur J Pharmacol. 1996;318:477–84. 9016941 10.1016/s0014-2999(96)00796-0

[45] Barron CC, Bilan PJ, Tsakiridis T, Tsiani E. Facilitative glucose transporters: Implications for cancer detection, prognosis and treatment. Metabolism. 2016;65:124–39. 26773935 10.1016/j.metabol.2015.10.007

[46] Huo X, Li J, Zhao F, Ren D, Ahmad R, Yuan X, et al. The role of capecitabine-based neoadjuvant and adjuvant chemotherapy in early-stage triple-negative breast cancer: a systematic review and meta-analysis. BMC Cancer. 2021;21:78. 33468087 10.1186/s12885-021-07791-y PMC7816481

[47] Cameron D. Lapatinib plus capecitabine in patients with HER2-positive advanced breast cancer. Clin Adv Hematol Oncol. 2007;5:456–8. 17679920

[48] Wang X, Wang SS, Huang H, Cai L, Zhao L, Peng RJ, et al. Effect of capecitabine maintenance therapy using lower dosage and higher frequency vs observation on disease-free survival among patients with early-stage triple-negative breast cancer who had received standard treatment: the SYSUCC-001 randomized clinical trial. JAMA. 2021;325:50–8. 33300950 10.1001/jama.2020.23370 PMC7729589

[49] Lin NU, Murthy RK, Abramson V, Anders C, Bachelot T, Bedard PL, et al. Tucatinib vs placebo, both in combination with trastuzumab and capecitabine, for previously treated ERBB2 (HER2)-positive metastatic breast cancer in patients with brain metastases: updated exploratory analysis of the HER2CLIMB randomized clinical trial. JAMA Oncol. 2023;9:197–205. 36454580 10.1001/jamaoncol.2022.5610 PMC9716438

[50] Lin NU, Borges V, Anders C, Murthy RK, Paplomata E, Hamilton E, et al. Intracranial efficacy and survival with tucatinib plus trastuzumab and capecitabine for previously treated HER2-positive breast cancer with brain metastases in the HER2CLIMB trial. J Clin Oncol. 2020;38:2610–9. 32468955 10.1200/JCO.20.00775 PMC7403000

[51] Peereboom DM, Alban TJ, Grabowski MM, Alvarado AG, Otvos B, Bayik D, et al. Metronomic capecitabine as an immune modulator in glioblastoma patients reduces myeloid-derived suppressor cells. JCI Insight. 2019;4:e130748. 10.1172/jci.insight.130748 PMC6948860 31600167

[52] Varma A, Mathaiyan J, Shewade D, Dubashi B, Sunitha K. Influence of ABCB-1, ERCC-1 and ERCC-2 gene polymorphisms on response to capecitabine and oxaliplatin (CAPOX) treatment in colorectal cancer (CRC) patients of South India. J Clin Pharm Ther. 2020;45:617–27. 32399998 10.1111/jcpt.13166

[53] Mackey JR, Mani RS, Selner M, Mowles D, Young JD, Belt JA, et al. Functional nucleoside transporters are required for gemcitabine influx and manifestation of toxicity in cancer cell lines. Cancer Res. 1998;58:4349–57. 9766663

[54] Marin JJ, Briz O, Rodríguez-Macias G, Díez-Martín JL, Macias RI. Role of drug transport and metabolism in the chemoresistance of acute myeloid leukemia. Blood Rev. 2016;30:55–64. 26321049 10.1016/j.blre.2015.08.001

[55] Nakano Y, Tanno S, Koizumi K, Nishikawa T, Nakamura K, Minoguchi M, et al. Gemcitabine chemoresistance and molecular markers associated with gemcitabine transport and metabolism in human pancreatic cancer cells. Br J Cancer. 2007;96:457–63. 17224927 10.1038/sj.bjc.6603559 PMC2360025

[56] Yamamoto M, Sanomachi T, Suzuki S, Uchida H, Yonezawa H, Higa N, et al. Roles for hENT1 and dCK in gemcitabine sensitivity and malignancy of meningioma. Neuro Oncol. 2021;23:945–54. 33556172 10.1093/neuonc/noab015 PMC8168817

[57] Deng T, Pan H, Han R, Huang D, Li H, Zhou L, et al. Gemcitabine sensitivity factors, hENT1 and RRM1 as potential prognostic biomarker for advanced biliary tract cancer. Int J Clin Exp Med. 2014;7:5041–9. 25664003 PMC4307450

[58] Achiwa H, Oguri T, Sato S, Maeda H, Niimi T, Ueda R. Determinants of sensitivity and resistance to gemcitabine: the roles of human equilibrative nucleoside transporter 1 and deoxycytidine kinase in non-small cell lung cancer. Cancer Sci. 2004;95:753–7. 15471562 10.1111/j.1349-7006.2004.tb03257.x PMC11158492

[59] Martín-Montalvo A, Villalba JM, Navas P, de Cabo R. NRF2, cancer and calorie restriction. Oncogene. 2011;30:505–20. 21057541 10.1038/onc.2010.492 PMC4684645

[60] Safdie F, Brandhorst S, Wei M, Wang W, Lee C, Hwang S, et al. Fasting enhances the response of glioma to chemo- and radiotherapy. PLoS ONE. 2012;7:e44603. 22984531 10.1371/journal.pone.0044603 PMC3439413

[61] Shi Y, Felley-Bosco E, Marti TM, Orlowski K, Pruschy M, Stahel RA. Starvation-induced activation of ATM/Chk2/p53 signaling sensitizes cancer cells to cisplatin. BMC Cancer. 2012;12:571. 23211021 10.1186/1471-2407-12-571 PMC3527202

[62] D’Aronzo M, Vinciguerra M, Mazza T, Panebianco C, Saracino C, Pereira SP, et al. Fasting cycles potentiate the efficacy of gemcitabine treatment in in vitro and in vivo pancreatic cancer models. Oncotarget. 2015;6:18545–57. 26176887 10.18632/oncotarget.4186 PMC4621909

[63] Elgendy M, Ciro M, Hosseini A, Weiszmann J, Mazzarella L, Ferrari E, et al. Combination of hypoglycemia and metformin impairs tumor metabolic plasticity and growth by modulating the PP2A-GSK3beta-MCL-1 axis. Cancer Cell. 2019;35:798–815 e5. 31031016 10.1016/j.ccell.2019.03.007

[64] Shen S, Iyengar NM. Insulin-lowering diets in metastatic cancer. Nutrients. 2022;14:3542. 10.3390/nu14173542 PMC9460605 36079800

[65] Vidali S, Aminzadeh S, Lambert B, Rutherford T, Sperl W, Kofler B, et al. Mitochondria: the ketogenic diet-a metabolism-based therapy. Int J Biochem Cell Biol. 2015;63:55–9. 25666556 10.1016/j.biocel.2015.01.022

[66] Vergati M, Krasniqi E, Monte GD, Riondino S, Vallone D, Guadagni F, et al. Ketogenic diet and other dietary intervention strategies in the treatment of cancer. Curr Med Chem. 2017;24:1170–85. 28093985 10.2174/0929867324666170116122915

