# Supplementary material for: GLUT3 enhances chemosensitivity in glioblastoma by transporting temozolomide and capecitabine
Source: Cell Death Discov. 2025 Aug 14;11:382. doi: 10.1038/s41420-025-02664-w (PMC12354831; doi:10.1038/s41420-025-02664-w)
Supplement: Supplementary file 1 — Table S1 [file 41420_2025_2664_MOESM1_ESM.doc]

Table S1. Information of the primers

| Oligonucleotides | Forward primer(5’-3’) | Reverse primer(5’-3’) |
| --- | --- | --- |
| Human β-actin-qRT-PCR | TGACGTGGACATCCGCAAAG | CTGGAAGGTGGACAGCGAGG |
| Human GLUT1-qRT-PCR | GGCCAAGAGTGTGCTAAAGAA | ACAGCGTTGATGCCAGACAG |
| Human GLUT2-qRT-PCR | GCTGCTCAACTAATCACCATGC | TGGTCCCAATTTTGAAAACCCC |
| Human GLUT3-qRT-PCR | GCTGGGCATCGTTGTTGGA | GCACTTTGTAGGATAGCAGGAAG |
| Human GLUT3-KD-qRT-PCR | TTAGATTACAGCGATGGGGAC | GGACCAGAGAGACGTGAGC |
| Human GLUT4-qRT-PCR | TGGGCGGCATGATTTCCTC | GCCAGGACATTGTTGACCAG |
| Human GLUT5-qRT-PCR | GAGGCTGACGCTTGTGCTT | CCACGTTGTACCCATACTGGA |
| Human GLUT6-qRT-PCR | CCGGACTACGACACCTTCC | GGATGTGTAGACCAGGGCATA |
| Human GLUT7-qRT-PCR | CAGTACGGCTACAACCTCTCT | TTGCGTGTCGCTCAAAGTAGG |
| Human GLUT8-qRT-PCR | CTAGTGGCCCCGGTCTACAT | CCGACGACGACCATTAGCTG |
| Human GLUT9-qRT-PCR | CCTCTACGGCTACAACCTGTC | AGAGTGTCTGGGTCTATTGGAC |
| Human GLUT10-qRT-PCR | CTTGCTGTATCTACGTGTCAGAG | CCAGCCAGTGCATAGTTGAGG |
| Human GLUT11-qRT-PCR | TTCACCAATGAGACATGGCAG | ACAGAGACACGATGAGGGACC |
| Human GLUT12-qRT-PCR | GAGGCTGCGGCATGTTTAC | CCAAGTTCATAACCCACCAGG |
| Human HMIT (GLUT13)-qRT -PCR | ACATTGCGGAGGTCTCACC | AGGCTCCATCAACAACACTTG |
| Human GLUT14-qRT-PCR | CTGCTCACGAATCTCTGGTCC | GCCTAATAGCACCGGCCATAG |
