# Supplementary material for: GLUT3 enhances chemosensitivity in glioblastoma by transporting temozolomide and capecitabine
Source: Cell Death Discov. 2025 Aug 14;11:382. doi: 10.1038/s41420-025-02664-w (PMC12354831; doi:10.1038/s41420-025-02664-w)
Supplement: Supplementary file 2 — Table S2 [file 41420_2025_2664_MOESM2_ESM.doc]

Table S2. Parameters of flow rates and gradients in LC-MS/MS analysis

| Time (min) | Module | Events | Parameter |
| --- | --- | --- | --- |
| 0.50 | Pumps | Pump B Conc. | 5 |
| 1.00 | Pumps | Pump B Conc. | 95 |
| 4.00 | Pumps | Pump B Conc. | 95 |
| 4.50 | Pumps | Pump B Conc. | 5 |
| 6.00 | Pumps | Pump B Conc. | 5 |
| 6.01 | System Controller | Stop | |
