# Supplementary material for: GLUT3 enhances chemosensitivity in glioblastoma by transporting temozolomide and capecitabine
Source: Cell Death Discov. 2025 Aug 14;11:382. doi: 10.1038/s41420-025-02664-w (PMC12354831; doi:10.1038/s41420-025-02664-w)
Supplement: Supplementary file 3 — Table S3 [file 41420_2025_2664_MOESM3_ESM.doc]

Table S3. Qualitative and quantitative ion pairs and mass spectrometric parameters in LC-MS/MS analysis

| Compound | Q1/Q3 Mass | DP (volts) | CE (volts) |
| --- | --- | --- | --- |
| TMZ | 195.200/138.200 | 56.000 | 14.000 |
| ^2^H_3_-TMZ | 198.200/138.200 | 56.000 | 14.000 |
| CAPE | 360.100/244.200 | 65.000 | 15.000 |
| ^2^H_11_-CAPE | 371.100/255.200 | 65.000 | 15.000 |
