# Supplementary material for: GLUT3 enhances chemosensitivity in glioblastoma by transporting temozolomide and capecitabine
Source: Cell Death Discov. 2025 Aug 14;11:382. doi: 10.1038/s41420-025-02664-w (PMC12354831; doi:10.1038/s41420-025-02664-w)
Supplement: Supplementary file 4 — Table S4 [file 41420_2025_2664_MOESM4_ESM.doc]

Table S4. Parameters of flow rates and gradients in the detection of TMZ and CAPE in tumor tissue

| Time (min) | Flow[ml/min] | B.Conc[%] | B.Curve |
| --- | --- | --- | --- |
| 0.00 | 0.3000 | 95.0 | 0 |
| 1.00 | 0.3000 | 95.0 | 0 |
| 1.50 | 0.3000 | 50.0 | 0 |
| 2.00 | 0.3000 | 50.0 | 0 |
| 3.00 | Pumps | 50.0 | 0 |
| 3.10 | Pumps | 95.0 | 0 |
| 6.00 | Pumps | 95.0 | 0 |
