# Supplementary material for: GLUT3 enhances chemosensitivity in glioblastoma by transporting temozolomide and capecitabine
Source: Cell Death Discov. 2025 Aug 14;11:382. doi: 10.1038/s41420-025-02664-w (PMC12354831; doi:10.1038/s41420-025-02664-w)
Supplement: Supplementary file 5 — Table S5 [file 41420_2025_2664_MOESM5_ESM.doc]

Table S5. Qualitative and quantitative ion pairs and mass spectrometric parameters in the detection of TMZ and CAPE in tumor tissue

| Compound | Q1/Q3 Mass | Dwell time | EP | Collision energy | CXP |
| --- | --- | --- | --- | --- | --- |
| TMZ-1 | 195.200/138.200 | 30 | 10 | 14 | 9 |
| TMZ-2 | 195.200/55.200 | 30 | 10 | 32 | 9 |
| ^2^H_3_-TMZ | 198.200/138.200 | 30 | 10 | 14 | 9 |
| CAPE-1 | 360.100/244.200 | 30 | 10 | 15 | 7 |
| CAPE-2 | 360.100/274.200 | 30 | 10 | 28 | 7 |
| ^2^H_11_-CAPE | 371.100/255.200 | 30 | 10 | 15 | 7 |
