# Supplementary material for: GLUT3 enhances chemosensitivity in glioblastoma by transporting temozolomide and capecitabine
Source: Cell Death Discov. 2025 Aug 14;11:382. doi: 10.1038/s41420-025-02664-w (PMC12354831; doi:10.1038/s41420-025-02664-w)
Supplement: Supplementary file 6 — Supplementary Figures and Legends [file 41420_2025_2664_MOESM6_ESM.docx]

**Supplement Figure 1**


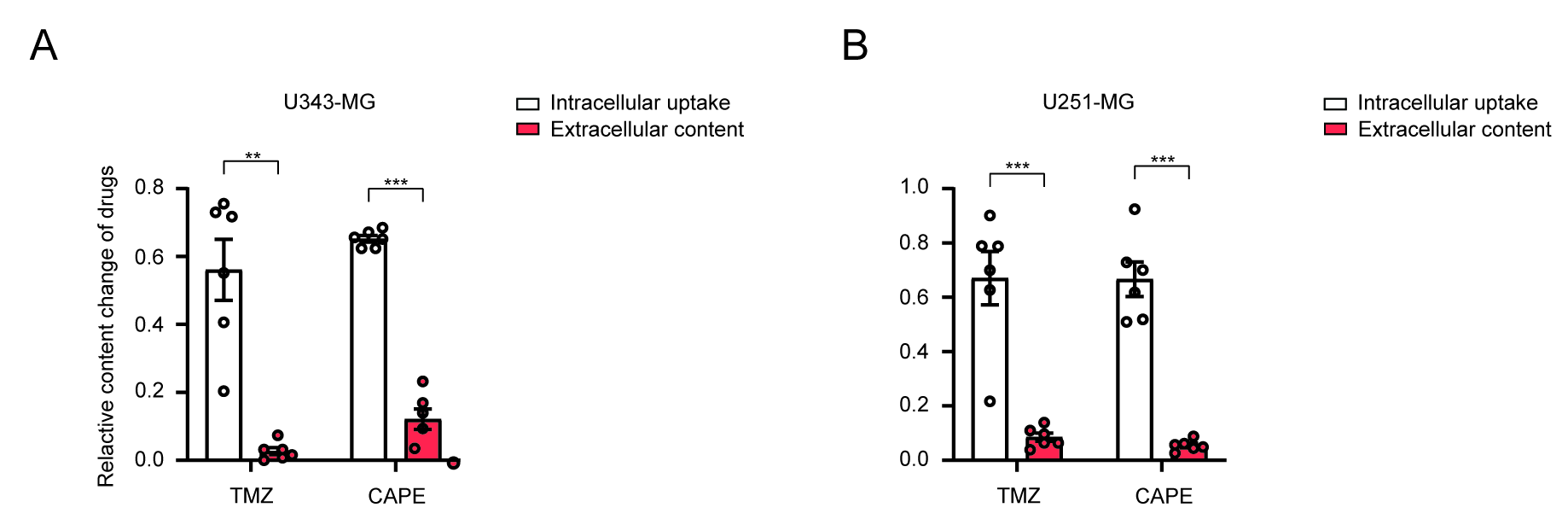


Comparison of extracellular and intracellular levels of TMZ and CAPE in U343-MG (A) and U251-MG (B) GBM cells. Data are presented as mean±SEM from five independent experiments. Independent-sample *t*-tests were used for statistical analysis using SPSS 20 (IBM). A *p*-value <0.05 was considered significant (* *P*<0.05, ** *P*<0.01, *** *P*<0.001).

**Supplement Figure 2**

**
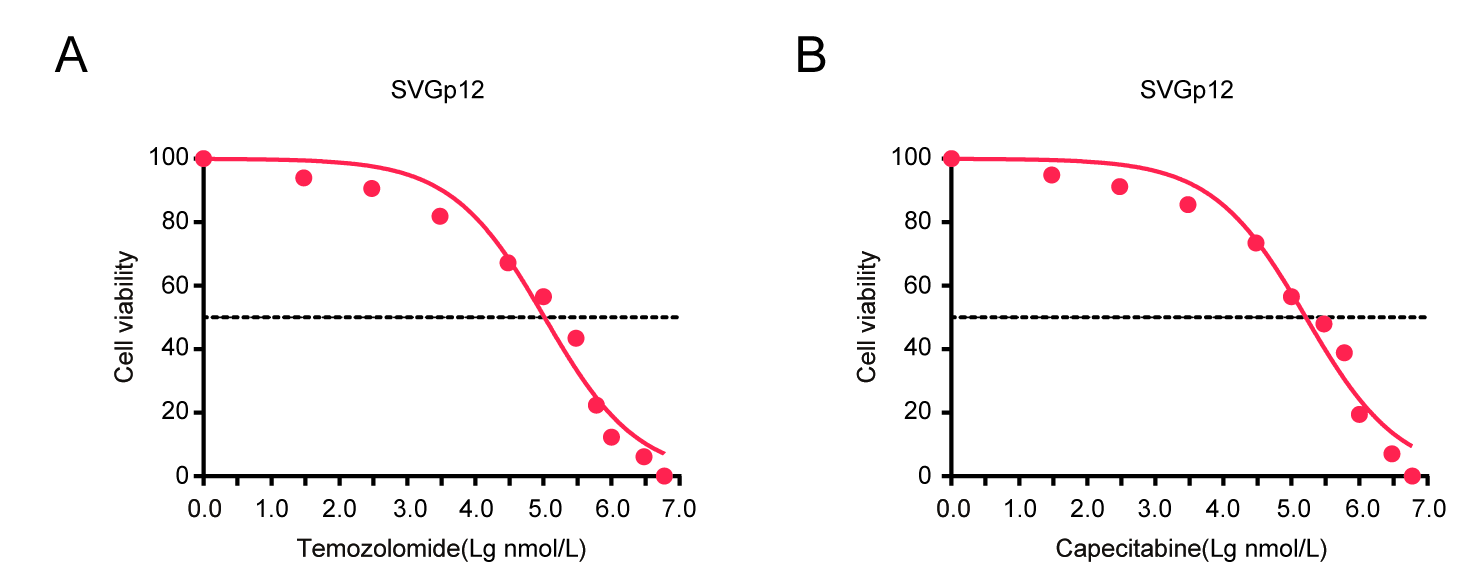
**

Effect of temozolomide (A) and capecitabine (B) on SVGp12 cell viability assessed by CCK-8 assay.

**Supplement Figure 3**


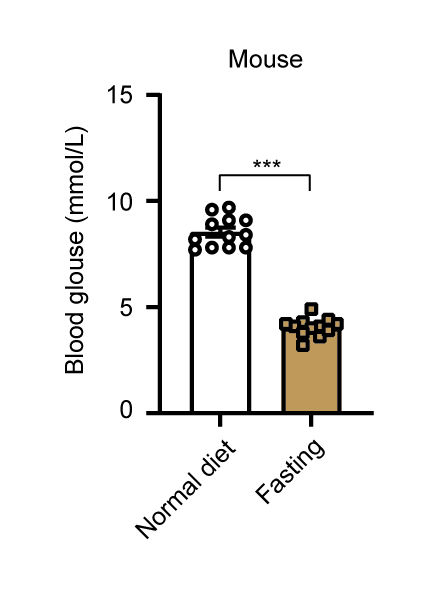


Fasting-induced dynamic changes in blood glucose levels in nude mice. Twenty-four age-matched BALB/c-nu nude mice (male: female=1:1) were randomly assigned to: control group (ad libitum-fe) and 24hr fasting group (water provided ad libitum). Blood glucose levels were measured at indicated time points via tail vein puncture using a calibrated glucometer (Yuwell 520). Data are presented as mean ± SEM from five independent experimental replicates. Statistical significance was determined by two-tailed unpaired Student's *t*-tests using SPSS 20 (IBM). Significant thresholds: * *P*<0.05, ** *P*<0.01, *** *P*<0.001.

**Supplement Figure 4**


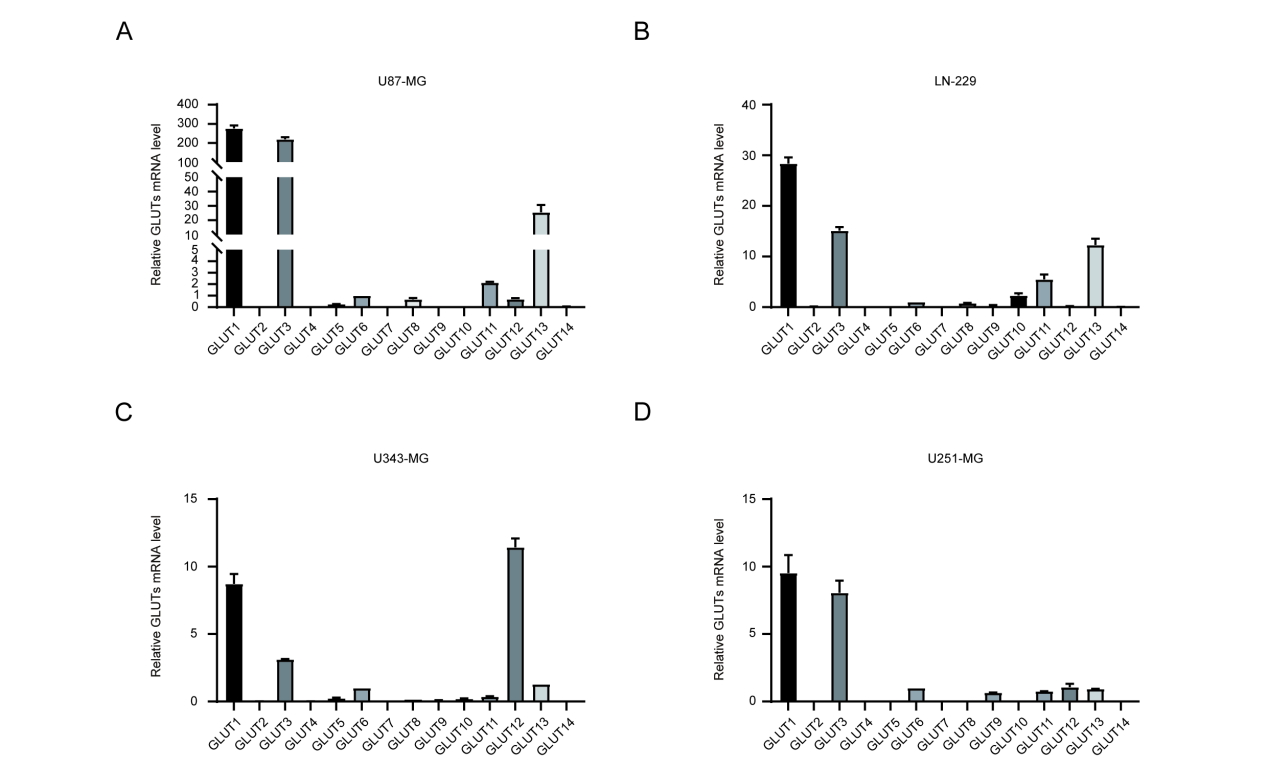


Comparative mRNA expression analysis of all 14 GLUT transporters (SLC2A1-14) across U87-MG (A), LN-229 (B), U343-MG (C), and U251-MG (D). Data are presented as mean ± SEM from five independent experiments.

**Supplement Figure 5**


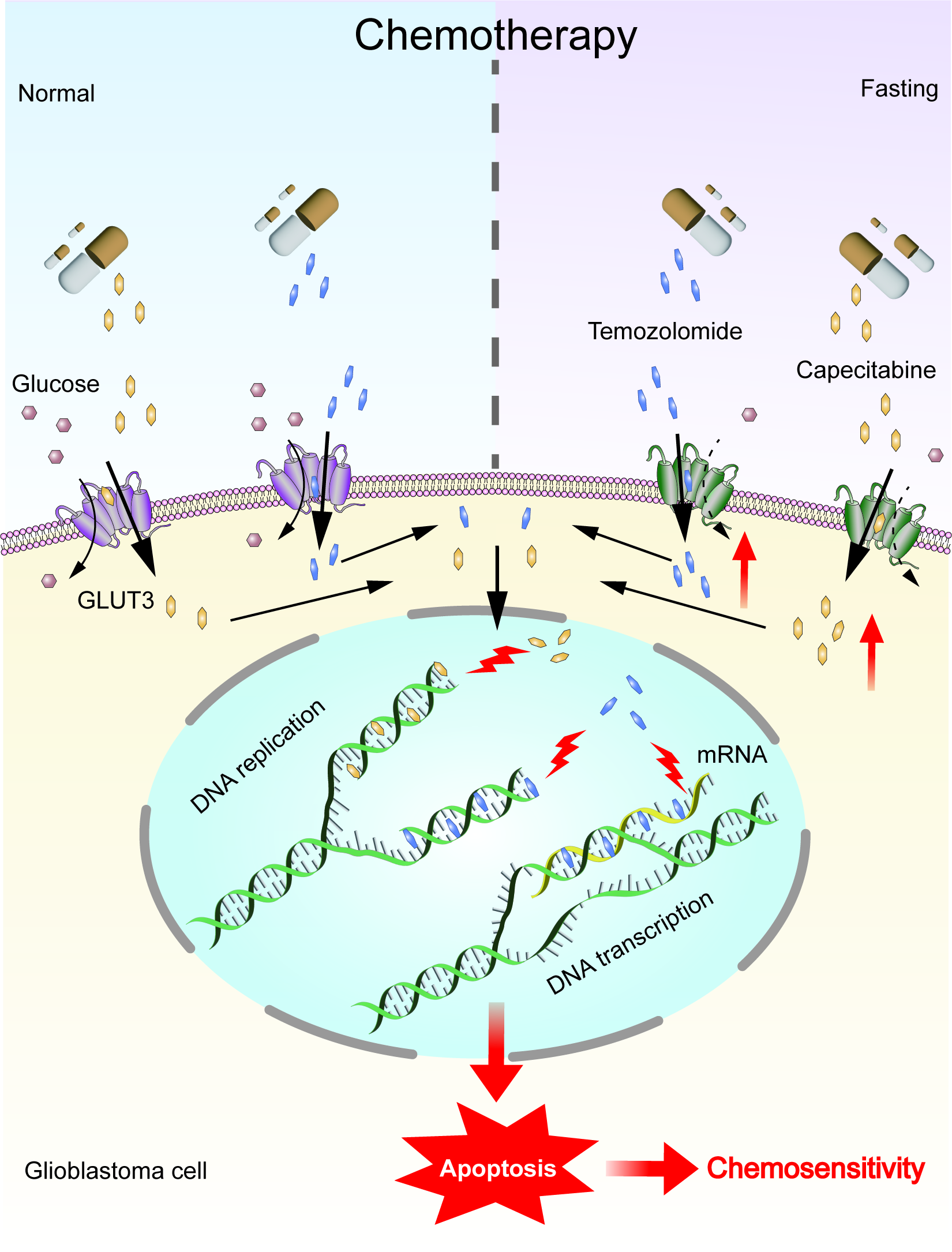


Mechanistic model of GLUT3-regulated chemosensitivity modulation in GBM.
