# Supplementary figures and images for: GLUT3 enhances chemosensitivity in glioblastoma by transporting temozolomide and capecitabine
Source: Cell Death Discov. 2025 Aug 14;11:382. doi: 10.1038/s41420-025-02664-w (PMC12354831; doi:10.1038/s41420-025-02664-w)

Figure 2

A

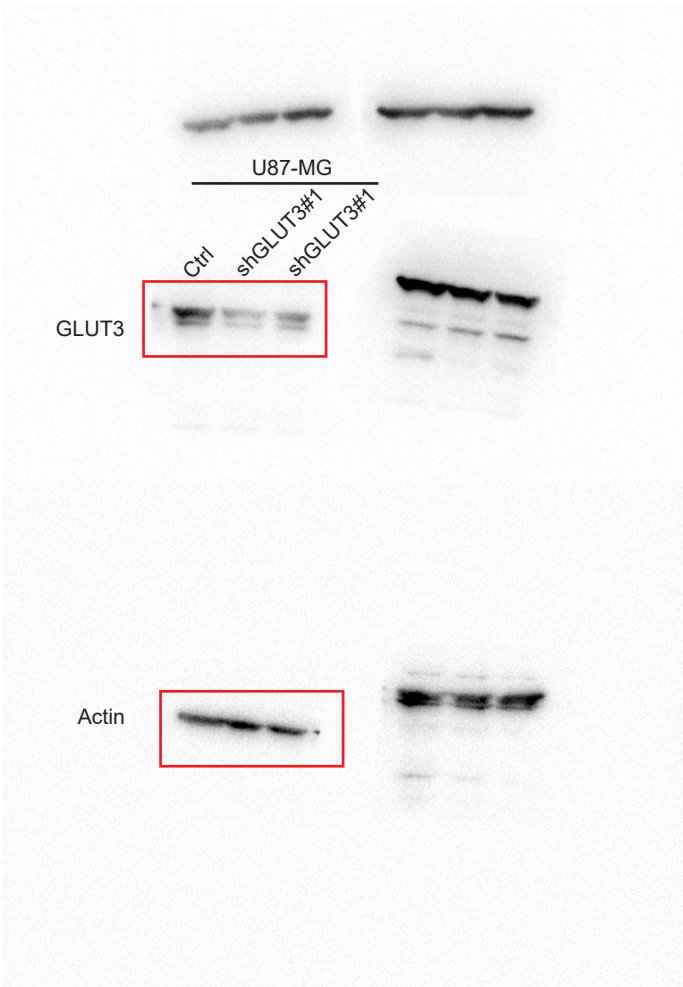

D

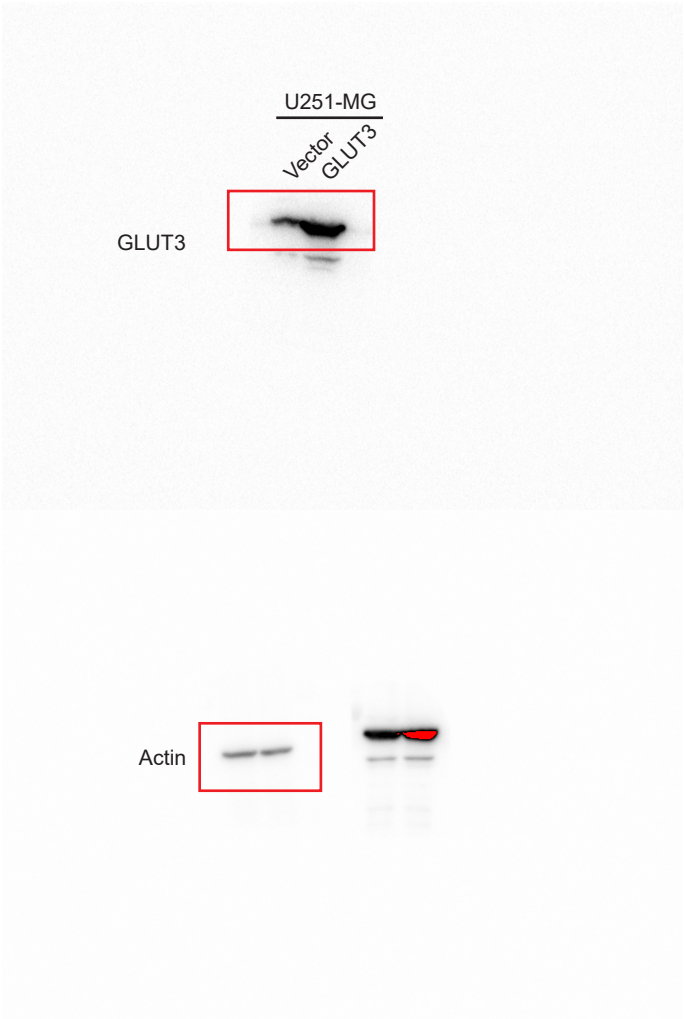

G

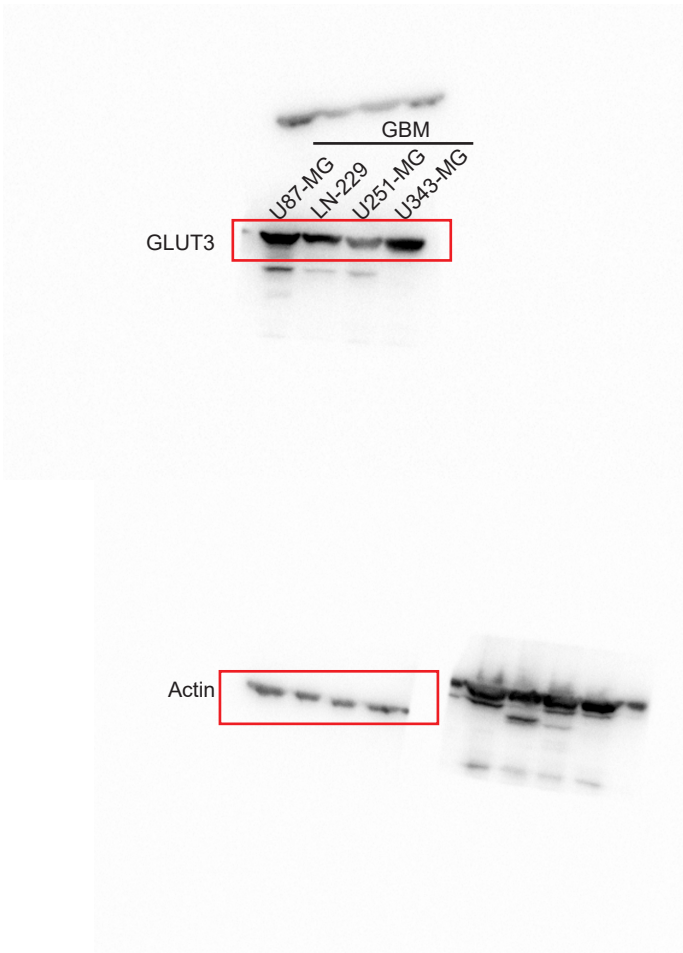

J-K

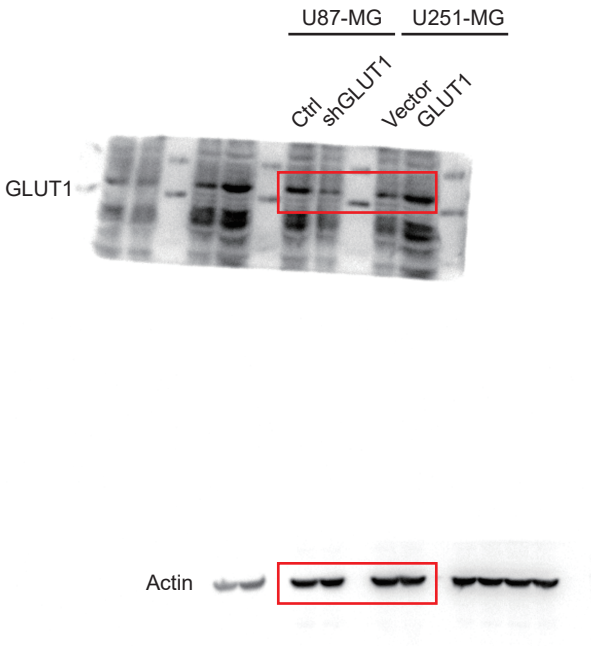

Figure 3

A

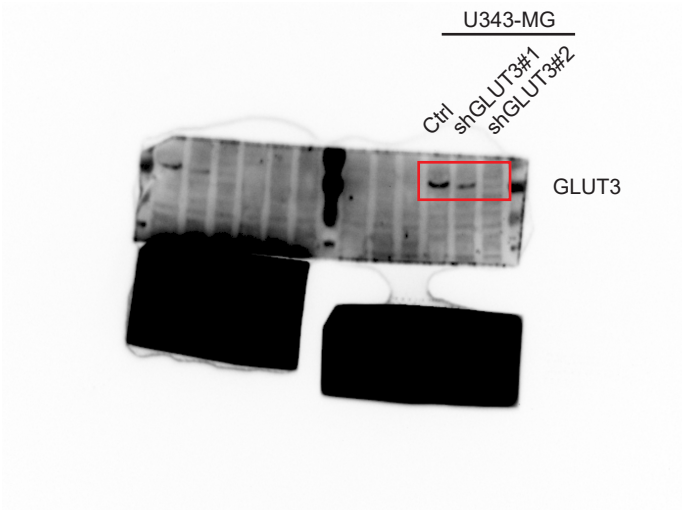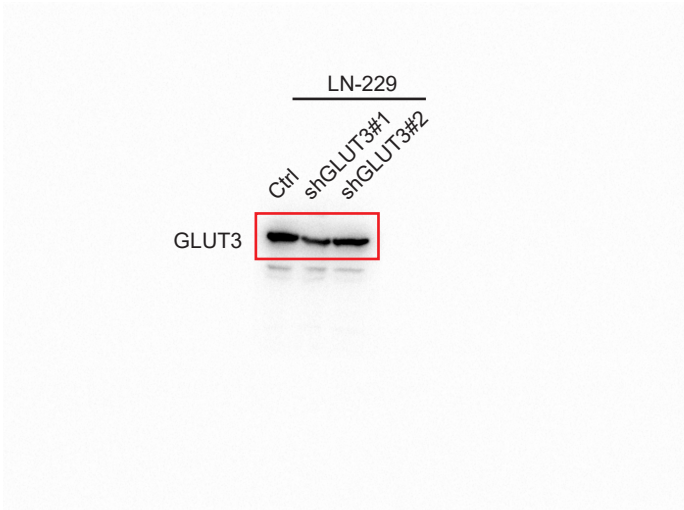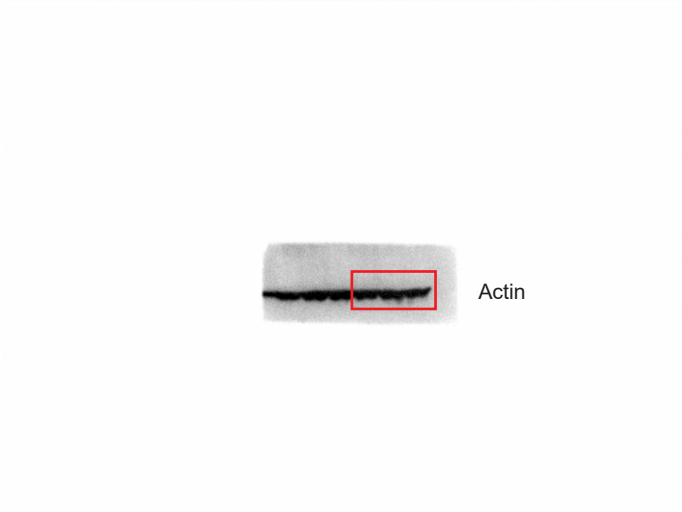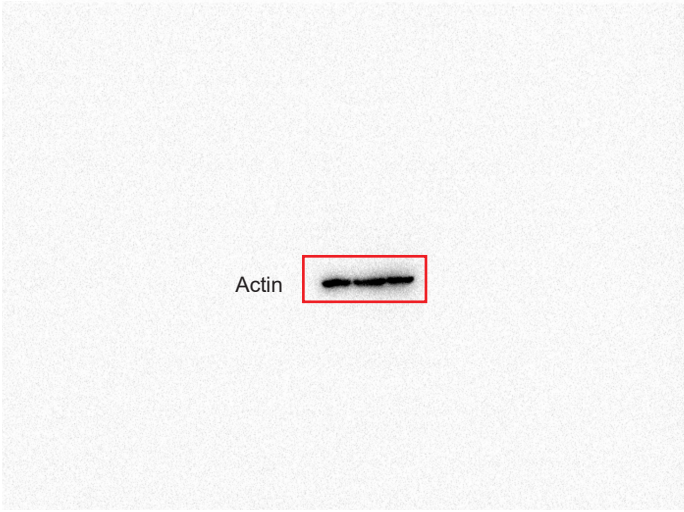

Supplement: Supplementary file 7 — Original Western blot [file 41420_2025_2664_MOESM7_ESM.pdf]
